# PNVCL-Based Multifunctional Nanogels Loaded with Curcumin, 5-Fluorouracil, and Gold Nanorods: Their Performance in Colon Cancer Cells

**DOI:** 10.3390/gels12010023

**Published:** 2025-12-25

**Authors:** Diana V. Félix-Alcalá, Mirian A. González-Ayón, Lizbeth A. Manzanares-Guevara, Alexei F. Licea-Navarro, Eugenio R. Méndez, Angel Licea-Claverie

**Affiliations:** 1 Centro de Graduados e Investigación en Química, Tecnológico Nacional de México/Instituto Tecnológico de Tijuana, Apartado Postal 1166, Tijuana 22454, Mexico; m21210020@tectijuana.edu.mx; 2 Departamento de Innovación Biomédica, Centro de Investigación Científica y de Educación Superior de Ensenada (CICESE), Ensenada 22860, Mexico; lizbeth.manzanaresg201@tectijuana.edu.mx (L.A.M.-G.); alicea@cicese.mx (A.F.L.-N.); 3 División de Física Aplicada, Centro de Investigación Científica y de Educación Superior de Ensenada (CICESE), Ensenada 22860, Mexico

**Keywords:** thermoresponsive nanogels, gold nanorods, photothermal therapy, colon cancer

## Abstract

This study presents the development and evaluation of multifunctional, thermoresponsive nanogels based on poly(*N*-vinylcaprolactam-*co-N*-vinylpyrrolidone) (P(NVCL-*co*-NVP)) with a poly(ethylene glycol) methyl ether methacrylate (PEGMA) shell and galactose (GAL) targeting ligand for colon cancer therapy. The nanogels were engineered to encapsulate two chemotherapeutic agents, curcumin (CUR) and 5-fluorouracil (5-FU), along with gold nanorods (GNRDs) to enable a synergistic chemo-photothermal treatment approach. These nanogels exhibit excellent biocompatibility and stability and a temperature-responsive drug release profile, leveraging the volume-phase transition temperature (VPTT) of the polymer network for controlled delivery. The inclusion of GNRDs permits efficient photothermal conversion upon near-infrared (NIR) irradiation, resulting in localized hyperthermia and, theoretically, improved cytotoxicity when combined with chemotherapeutics. In vitro studies on colon cancer cells demonstrated enhanced drug accumulation, photothermal ablation when the GNRD concentration was above a threshold, and superior antitumor efficacy of the CUR/5-FU-loaded systems. The effectiveness of the chemo/photothermal combination could not be demonstrated, possibly due to the low concentration of GNRD and/or the use of a single irradiation step only. This work highlights the potential of P(NVCL-*co*-NVP):PEGMA:GAL nanogels as versatile nanocarriers for combined chemo-photothermal therapy. A more effective chemo/photothermal combination for colon cancer treatment can be achieved through the optimization of the GNRD loading/irradiation dosage.

## 1. Introduction

Colon cancer is ranked as the fifth most fatal type of cancer worldwide [1]. Colon cancer treatment typically consists of chemotherapy with 5-fluorouracil (5-FU) individually or in combination with folinic acid, irinotecan, and oxaliplatin (FOL-FOX). Curcumin (CUR) is a polyphenol of the rhizome *Curcuma longa* L.; CUR has shown therapeutic potential for its antioxidant, anti-inflammatory, and antitumor properties [2,3,4]. Likewise, CUR has been reported as an adjuvant to chemotherapeutic agents in colon cancer [5,6,7]. Clinical studies have evaluated patients with metastatic colon cancer using the combined treatment of FOL-FOX with different doses of CUR, reporting a greater tolerance of patients to chemotherapy due to simultaneous administration [5]. However, the bioavailability of CUR is limited by its poor solubility but can be improved in nano-formulated systems [8,9].

Chemotherapy destroys growing cells indiscriminately, affecting both cancerous and normal cells. In contrast, targeted therapy allows for the accumulation of drugs in the desired tissue. Nanocarriers can transport and protect drugs, enhance the solubility of hydrophobic chemotherapeutic agents, and enable the targeting of tumors without harming healthy tissues, thereby overcoming the negative effects of chemotherapy [10]. Responsive “smart” polymers have been widely used for the preparation of nanocarriers with application in cancer treatment [11,12]; smart polymers can respond reversibly to an external stimulus from the medium, such as pH [9,13,14], light intensity [15,16], magnetic field [17,18], or temperature [19,20,21]. A temperature-responsive polymer is poly(*N*-vinylcaprolactam) (PNVCL), which has potential applications in controlled drug release, as certain pathological conditions can exhibit an increase in local temperature [22]. Additionally, PNVCL is biocompatible, and its response temperature in water is close to physiological values (30–34 °C) [23]. Thermoresponsive nanocarriers of interest are nanogels (NGs) [24,25]; an NG are a nanometric three-dimensional crosslinked polymer that shows properties such as biocompatibility, desirable mechanical properties, and an interior network for multiple molecule incorporation [24,26,27]. At an increment of temperature, thermoresponsive three-dimensional NGs exhibit a volume-phase transition temperature (VPTT); changes are quick and reversible [28]; as a consequence, the loaded chemotherapeutic agents can be easily released from the network. A combination of polymers is generally used for the preparation of thermoresponsive core–shell NGs with tailored properties, for example, NGs of poly(*N*-isopropyl methacrylamide) (PNIPMAM) shell and core of poly(*N*-isopropyl acrylamide) (PNIPAM) [20], NGs of poly(2-diethylaminoethyl methacrylate-co-*N*-vinylcaprolactam) (P(DEAEMA-co-NVCL)) as core, and poly(ethylene glycol) methyl ether methacrylate) (PEGMA) as shell [14] as well as NGs of poly(*N*-vinylcaprolactam-co-*N*-vinyl pyrrolidone) (P(NVCL-co-NVP) as core and PEGMA as shell [19]. Combinations of individual characteristics of each component enhance their physical, chemical, and/or biological properties of the NGs, altering the VPTT, compartmentalizing the thermal response, and adding additional responses/clues. For example, a polyetilenglicol (PEG) shell protects the NGs from aggregation and prevents recognition and opsonization by the mononuclear phagocytic system, resulting in prolonged systemic circulation after intravenous injection [29,30]. In addition to the chemistry of thermoresponsive NGs, incorporating biological molecules into NGs could improve affinity to cancerous cells through overexpressed receptor molecules on the extracellular surface, mediating NGs uptake into cancer cells [31,32]. Some biomolecules can be attached to nanocarrier polymers for targeting, such as different types of carbohydrates; for instance, mannose [33], glucose [34,35], and galactose (GAL) [36,37] have been reported. GAL has shown affinity for receptors such as the pro-metastatic protein galectin-3 (GAL-3), and GAL-3 is overexpressed in colon cancer [38,39], so GAL is a valid clue for improving the affinity of NGs to colon cancer cells.

Hyperthermia is an alternative therapy that exposes target tissue to elevated temperatures to either induce cellular susceptibility (41–45 °C) or achieve thermal ablation at temperatures above 47 °C. One such modality in cancer treatment is photothermal therapy, in which nanoparticles convert light energy into heat to induce necrosis or apoptosis in cancer cells [40]. Certain metals, such as gold, reduced to the nanoscale, exhibit a phenomenon called localized surface plasmon resonance (LSPR). Gold nanoparticles, due to their stability, biocompatibility, and optical properties derived from their interaction with light, are of significant interest for biomedical applications [40,41,42]. Particularly when fabricated in specific geometries like rods, e.g., gold nanorods (GNRDs), the LSPR of GNRDs can be tuned to near-infrared (NIR) wavelengths depending on their aspect ratio (length to thickness). The importance of a LSPR in the NIR region (wavelength range from 800 to 1200 nm) is a deeper penetration of incident light into biological tissues with minimal absorption [43,44]. A combination of hyperthermia with chemotherapy can enhance the sensitivity of cancer cells to specific chemotherapeutic agents, potentially improving local drug release; while the degree of sensitization depends on various factors, including the type and concentration of the drug, the tumor type, the temperature increases within the tumor, and the timing between light irradiation elapses [45,46,47]. Studies have shown that combined localized hyperthermia can enhance the cytotoxicity of chemotherapeutic agents [42,48]. The effect is closely tied to the thermal dose, with the maximum response observed at temperatures between 40 °C and 43 °C [49].

**Figure 1:**
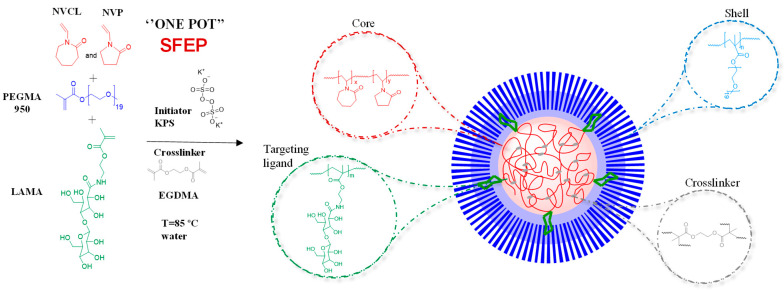
Schematic representation of a P(NVCL-co-NVP):PEGMA core–shell NGs containing GAL-units: Monomer units in the core are colored red, monomers in the shell are colored blue and monomers in the core-shell interface are colored green.

The aim of this report was to explore the suitability and limits of P(NVCL-*co*-NVP) core NGs with galactose targeting the ligand interface and a PEGMA shell (Figure 1) as a nanocarrier of 5-FU and CUR and also be able to load GNRDs for controlled delivery of the active compounds in a combined chemo-photothermal therapy to colon cancer cells.

## 2. Results and Discussion

### 2.1. The Nanocarriers: Functionalized Nanogels

The synthesis of thermoresponsive and galacto-functionalized NGs as nanocarriers was achieved by Surfactant Free Emulsion Polymerization (SFEP); this method allows the preparation of NGs with core–shell morphology based mainly on the hydrophobic and hydrophilic character of the monomers under the reaction conditions. At the reaction temperature, the PNVCL units are hydrophobic and will remain in the core of the NGs, while PEGMA is hydrophilic and will form a shell stabilizing the core. In addition, according to previous reports, PNVCL-based thermoresponsive nanogels exhibit a VPTT-type phase transition governed by the balance between hydrophilic interactions with water and interchain hydrophobic associations. The incorporation of hydrophilic comonomers such as *N*-vinylpyrrolidone (NVP), PEGMA, and galactose-functionalized unit (LAMA) systematically modifies this thermal response. In particular, NVP increases water affinity through hydrogen bonding, shifting the VPTT to higher temperatures. Similarly, PEGMA, due to its highly hydratable PEG side chains and steric effect, raises the transition temperature and enhances colloidal stability, allowing fine-tuning of the thermal response, especially in nanogels and core–shell systems. Meanwhile, the introduction of GAL increases the overall hydrophilicity of the system due to the abundance of hydroxyl groups, which also tends to elevate the VPTT, while conferring biological functionality (e.g., cellular recognition) without suppressing the thermal response. Altogether, these comonomers act as modulators of hydration and the macromolecular architecture of PNVCL, enabling the adjustment of thermal transition to specific ranges, including physiological ones, and broadening their applicability in smart and biomedical systems, such as drug delivery systems [19,37]. In this work, the goal was to obtain nanocarriers in sizes around 200 nm, with a response temperature between 38 and 40 °C, provided with a PEG shell and containing GAL moieties for targeting purposes.

**Figure 2:**
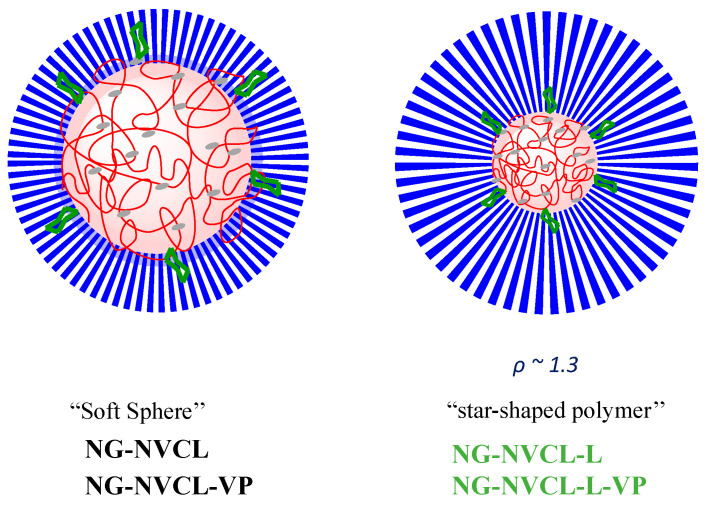
Theoretical morphology of NGs in water based on ρ-parameter by light scattering data: The core is colored red, the shell is blue and the core-shell interface is green.

Nanogel composition was determined by ^1^H-NMR (Figures S1–S4, Table 1). The composition data indicated preferred incorporation of PEGMA and NVCL into NGs compared to NVP or LAMA. Galacto-functionalized NGs incorporated LAMA greater than 10 mol% and a PEGMA segment superior to 32 mol%; PEGMA and LAMA improve NGs hydrophilicity, and PEGMA forms a biocompatible shell to be able to go unnoticed by the immune system. On the other hand, data obtained through light scattering methods provided NGs characteristics like molecular weight (M_w_), radius of gyration (R_g_), and the second virial coefficient (A_2_) obtained by SLS (Figures S5–S8) and the hydrodynamic radius (R_h_) by DLS (Figure S10a). NGs show high M_w_ values ranging from 2.01 × 10^6^ to 1.4 × 10^8^ g mol^−1^ (Table 1). Values of A_2_ are related to a good interaction between NGs and water. Additionally, the ρ parameter is obtained by the ratio between R_g_ and R_h_; this parameter relates to the morphology of the polymeric systems: obtained ρ values were from 0.92 to 1.37. Reports in the literature associate spherical micelle-type morphologies of flexible polymers “soft sphere” with values around ρ = 1 (NG-NVCL and NG-NVCL-VP, larger nanogels 140–260 nm in diameter), while the values obtained for GAL-functionalized NGs (NG-NVCL-L and NG-NVCL-L-VP, smaller NGs ~80 nm in diameter) of around ρ = 1.3 resemble a “star-shaped polymer” (Figure 2) [50].

NGs were further characterized in PBS at pH 7.4, pH 6.8, and pH 5 by DLS to determine the behavior under simulated physiological conditions for in vitro drug release experiments (Figure S9). NGs containing NVP or LAMA exhibited unimodal size distributions, with hydrodynamic diameters (D_h_) between 86 and 280 nm and a polydispersity index (PDI) from 0.219 to 0.273; in general, no size changes in NGs were observed when the pH varied from 7.4 to 5, as expected for PNVCL/PEGMA NGs since they are constructed using non-ionic polymers [22].

### 2.2. Thermoresponsive Behavior and Zeta Potential of Nanogels

**Figure 3:**
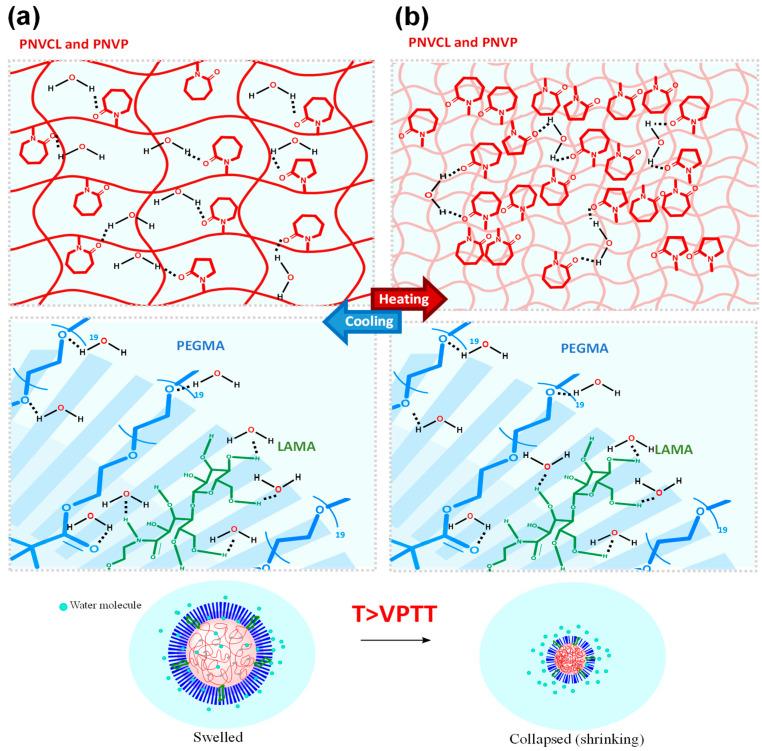
Representative scheme of hydrogen bond formation between water molecules and the nanogel in the core and in the shell: (**a**) below the VPTT, and (**b**) above the VPTT.

Temperature-sensitive polymer networks shrink above the VPTT and expand below this value. Below the VPTT, PNVCL-based nanogels adopt hydrated coil conformations stabilized by polymer–water hydrogen bonding, primarily involving lactam amide groups. PNVP strengthens the hydration shell through carbonyl–water interactions, while PEGMA provides additional steric and enthalpic stabilization via its ethoxylated side chains. On the other hand, in LAMA-containing systems, galactosyl moieties introduce highly structured hydrogen-bond networks, delaying thermal dehydration. As temperature increases toward the VPTT, these interactions are progressively disrupted, releasing structured water molecules and shifting the thermodynamic balance toward polymer–polymer interactions. This dehydration process is entropically driven and only partially counteracted by PEGMA and LAMA. Finally, above the VPTT, a cooperative coil to globule transition occurs, dominated by hydrophobic association of dehydrated PNVCL segments, leading to nanogel collapse and formation of transient physical crosslinks. PNVP contributes secondary dipolar interactions, while partially dehydrated PEGMA segments may engage in weak thermally induced associations (Figure 3) [51].

**Figure 4:**
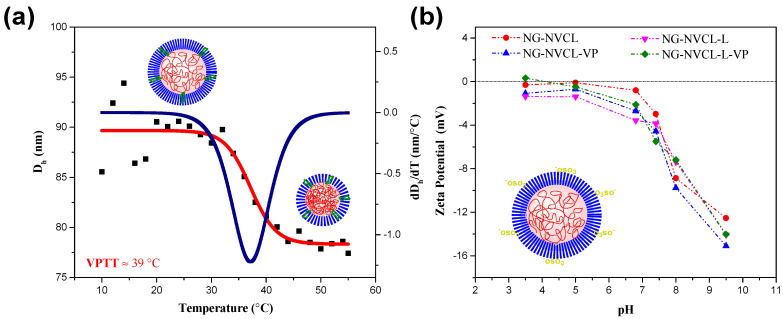
Thermoresponsive behavior diagram of NG-NVCL-L-VP in PBS pH 7.4 (**a**) and zeta potential of NGs at different pH values (**b**). In subsection (**a**) the squares are the measured values while a trend line (red) was added, and the blue line is the first derivative of the trend line.

The thermosensitive behavior of nanogels was analyzed by DLS at pH 7.4 and pH 5. VPTT values are summarized in Table 2. The minimum of the first derivative, ΔD_h_/ΔT, was taken as the VPTT. The nanogel NG-NVCL exhibited a VPTT close to 32 °C at pH 7.4 (Figure S10a), which is consistent with previous reports for nanogels based on PNVCL and PEGMA, where the VPTT is around 30–36 °C [52]. However, this value increased until 37 °C, when 15 mol% of LAMA was included (Figure S10g), and shifted to 39 °C if, additionally, 22 mol% of VP was added (Figure 4a). The addition of the comonomer (NVP or LAMA) causes an increase in the VPTT, as expected from their hydrophilic character, helping to shift the VPTT close to the physiological temperature. On the other hand, in PBS pH 5, VPTT values shifted to higher values for NG-NVCL and NG-NVCL-VP (the larger NGs), 45 and 51 °C, respectively (Figure S10c,f), while the VPTT for GAL-functionalized nanogels (smaller NGs) did not change but is observed at less sigmoidal behavior at this pH value (Figure S10i,l).

At lower pH of the medium (higher proton concentration), the electrostatic repulsion or attraction forces between the polymer chains change and formation of additional hydrogen bonds that could affect solubility and generate resistance to collapse may appear; partial ionization of NVP/NVCL units as compared to pH 7.4 may alter the swelling of the large core NGs; the result is that not all hydrogen bonds to water are broken by calorific energy up to 51 °C, and the process requires an additional increase in temperature.

Additionally, the dependence of zeta potential on the pH of NG was evaluated (Figure 4b). NG exhibits negative values of zeta potential and approaches zero at low pH. At the pH interval of analysis, NG-NVCL and NG-NVCL-L-VP nanogels approach the isoelectric point at around pH 4. Since PNVCL, PEGMA, and PNVP are neutral, arising from non-ionizable monomers, this pH dependence cannot be attributed to intrinsic ionization of the polymer network. Consistent with previous reports, the negative surface charge observed at basic medium is primarily associated with residual anionic groups derived from the persulfate initiator, which remain bound to the nanogel surface and are more strongly expressed under neutral–alkaline conditions [53]. In nanogels incorporating a galactose-derived monomer (LAMA), an additional contribution to negative surface charge under neutral–alkaline conditions has been reported, attributed to both monomer chemistry and local microenvironmental effects [37]. Other studies of galacto-functionalized nanoparticles have also shown negative zeta potential at pH 7.4 [54,55]. Overall, these results indicate that the pH-dependent zeta potential behavior of PNVCL–PEGMA nanogels arises from a weak, indirect surface charge, dominated by initiator residues and ionic environment rather than polyelectrolyte-like behavior.

### 2.3. Loading of Two Drugs: 5-FU and CUR and Loading of GNRDs in the Nanogels

**Figure 5:**
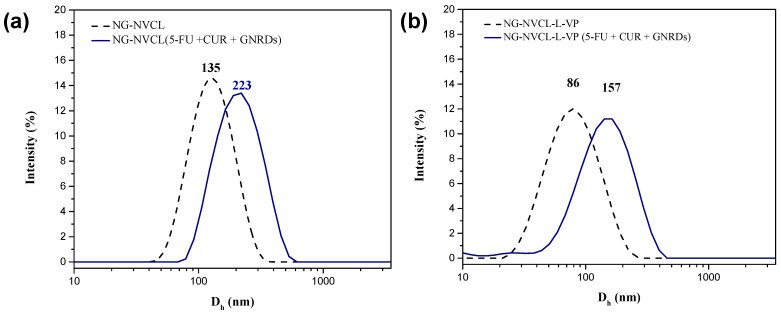
Size distribution by DLS in PBS 7.4 before and after concurrent loading of 5-FU, CUR, and GNRDs of (**a**) NG-NVCL and (**b**) NG-NVCL-L-VP.

In general, it is expected that the NGs increase in size when loaded simultaneously with 5-FU, CUR, and GNRDs (compare Table 1 and Table 3); there are also geometrical constraints to the loading of GNRDs related to their relatively “large” size. Figure 5 compares non-loaded and loaded NG-NVCL and NG-NVCL-L-VP size distributions; an increase in average diameter from 65 to 82% and PDI to values greater than 0.39 was observed, indicating a size increase accompanied by a decrease in size homogeneity of loaded NGs with respect to unloaded NGs. A TEM micrograph of this deformed nanogel loaded with GNRDs can be seen in Figure S11. The other NGs are shown in Figure S12.

The *DLC* and *DLE* of 5-FU and CUR in NGs were determined, as shown in Table 3. For 5-FU, the *DLC* was between 21 and 38% by weight, and *DLE* was between 25 and 40%. Several loading percentages of 5-FU have been reported in nanocarriers in the literature, with percentages between 10 and 20% [19,56] and also up to 36% [57]. On the other hand, NGs loaded with CUR were able to load only from 2 to 3% CUR, with efficiencies around 20%. The higher loading of 5-FU with respect to CUR in nanogels can be attributed to the fact that 5-FU is a smaller molecule and has good solubility in water at concentrations below 13 mg mL^−1^, while CUR is very hydrophobic and a relatively bulky molecule; therefore, it is difficult to encapsulate it in the already 5-FU-loaded nanogels. Studies of loading CUR only in other kinds of nanogels have shown higher *DLC* from 4% to 18% [9,58].

**Figure 6:**
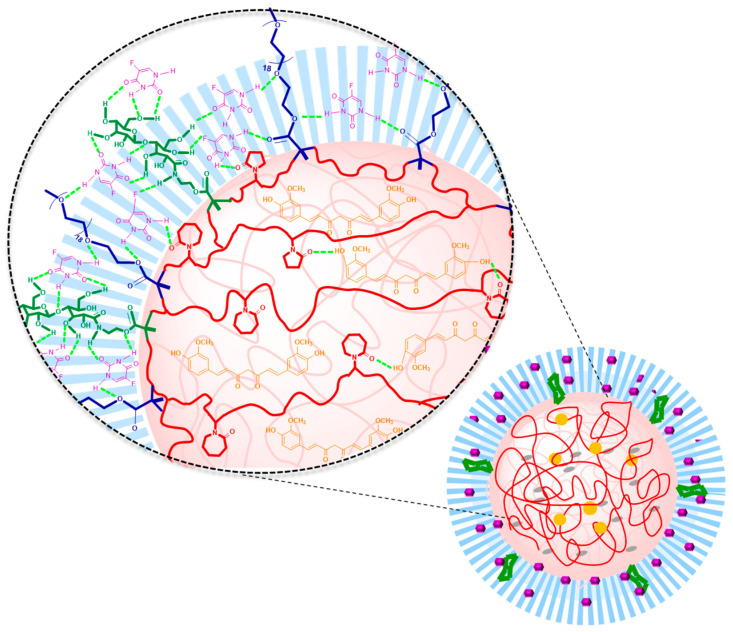
Schematic representation of possible interactions of 5-FU and CUR with the nanogel containing LAMA: 5-FU is colored violet, CUR is colored orange, core units are red and shell units are colored blue; LAMA units are dark green and hydrogen bonding interactions light green.

In addition, the loading of 5-FU involves the formation of hydrogen bonds with the whole polymeric network, like the amide of NVCL or NVP on the core and the ester group of PEGMA in the shell, while CUR is expected to interact only with the core of the NG by hydrophobic and Van der Waals forces (Figure 6). In addition, CUR can form hydrogen bonds with both OH groups in the carbonyl of NVCL and NVP. The highest *DLC* for 5-FU was for NGs containing also LAMA, NG-NVCL-L, and NG-NVCL-L-VP with 32 and 35%, respectively. This is attributed to the carbohydrate group of GAL, the amount of oxygen, and OH groups in the LAMA structure, which intensified the formation of hydrogen bonds with 5-FU (Figure 6).

**Figure 7:**
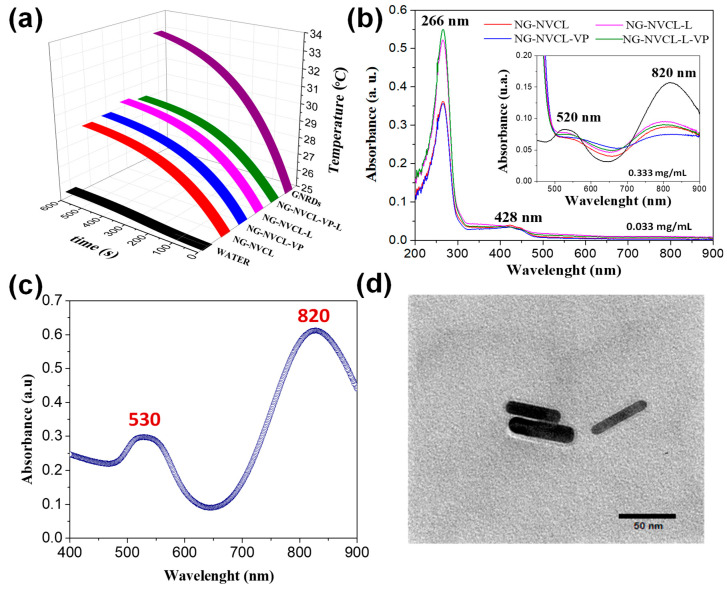
NGs loaded with 5-FU, CUR, and GNRDs. (**a**) Temperature increasing of NG (1.6 mg mL^−1^) compared to water and GNRDs; (**b**) UV-vis spectrum of loaded NG and GNRDs; (**c**) UV-vis spectrum of GNRDs; (**d**) TEM micrograph of GNRDs.

The loaded NGs were analyzed by TGA to determine the loading of GNRDs. The gold percentage (%*Au*) was obtained with Equation (3) through the ratio between the residue at 550 °C for pure NGs (Figure S13) and loaded NGs (Figure S14); the results are reported in Table 3. Figure S13b represents the thermograms for loaded NG-NVCL-VP; two stages of weight loss are shown: the first decomposition temperature (T_d_), with an average temperature at 265 °C (first peak maximum), is associated with the loaded chemotherapeutic agents, probably 5-FU in the greatest quantity. The literature associates a weight loss step for 5-FU starting at 200 °C [59]. The second T_d_ with an average temperature at 418 °C (maximum of second peak) is associated with the main nanogel decomposition step. For NG-NVCL-L-VP loaded with 5-FU, CUR, and GNRDs, a residue of 10.5 wt% remained; the residue was obtained from the thermogram of unloaded NG-NVCL-VP (4.17 wt%), therefore, around 6% corresponds to the remaining gold. The percentage of gold (%*Au*) was ≤10% for all NGs and is associated with the heating effect expected when the GNRD-loaded NGs are irradiated with a NIR laser. Depending on the size of the nanogel and surface charge, NG can trap only one or a few GNRDs; the GNRDs tend to become embedded between the polymeric network or be retained superficially by electrostatic interactions. The temperature increases in NG in PBS solutions (1.6 mg mL^−1^) under NIR irradiation were evaluated; the nanogels were compared with a solution of GNRDs (10% *v*/*v*) and deionized water as a blank (Figure 7a). The measuring system consisted of a foam-isolated beaker containing a thermocouple where the sample was poured. The NIR laser was located 10 cm above the sample, and the thermocouple registered the temperature every 10 s. The solution of GNRDs diluted in water increased the temperature by 8 °C in 10 min. It is observed that the performance of the NGs loaded with GNRDs was not as good; this is due to their content of GNRDs (6–10% gold). NG-NVCL-L was the nanogel with the highest percentage of gold (10%) and achieved a slightly higher temperature increase than the other NGs. It is observed that the nanogels increased the temperature by around 3 °C at 5 min and approximately 5 °C at 10 min. Although the temperature increase may be considered small, it may be sufficient, under the study conditions, for surpassing the VPTT. It is expected that, with NIR laser irradiation, the nanogel systems will be able to overcome their VPTT and reach temperatures of 40 °C or slightly higher, enough to sensitize the target cells to chemotherapy with 5-FU but not enough for hyperthermia.

Figure 7b shows the UV-vis spectrum of GNRDs and loaded nanogels. Peaks at 266 and 428 nm correspond to the absorbance of 5-FU and CUR, respectively (Figures S15 and S16). Furthermore, two absorbance peaks are observed related to GNRDs at 520 and 820 nm, corresponding to the typical absorbance signals for transversal and longitudinal LSPR in GNRDs (Figure 7c,d). Particularly, the absorbance peak close to 800 nm (NIR region) is of great interest for biomedical applications, due to the optimal light penetration and minimal absorption by tissue chromophores and water at this wavelength, making the loaded NGs suitable for photothermal therapy [43,60].

### 2.4. Controlled Release of 5-FU and CUR from Nanogels Without NIR Irradiation

**Figure 8:**
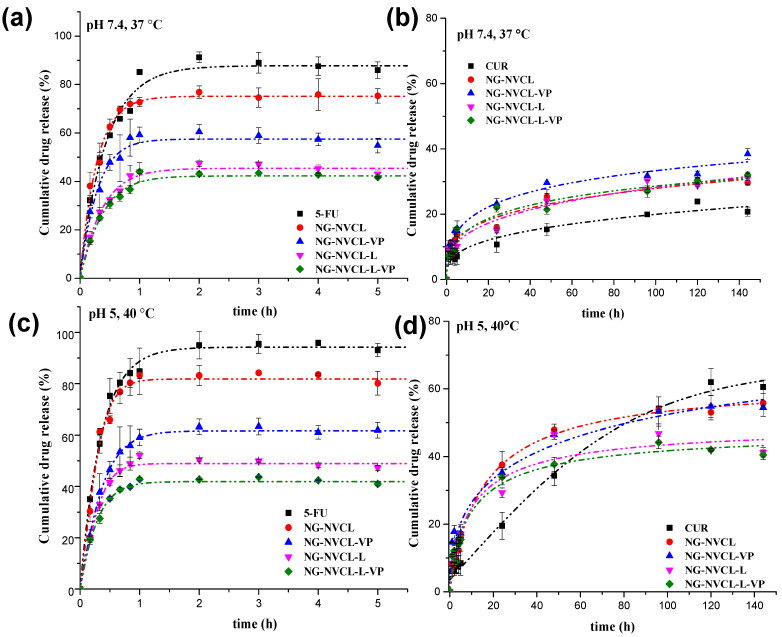
Release profiles of 5-FU and CUR at pH 7.4, 37 °C (**a**,**c**), and at pH 5, 40 °C (**b**,**d**), through nanogels as well as free 5-FU and CUR.

In vitro release studies of chemotherapeutic agents were carried out mimicking the normal physiological conditions (pH 7.4, 37 °C) and slight hyperthermia and endosomal conditions (pH 5, 40 °C) throughout 24 h for 5-FU and 144 h (6 days) for CUR. Release profiles were compared to nanocarrier free agents (5-FU or CUR). The profiles are shown in Figure 8; for 5-FU, only the first 5.5 h are plotted for clarity since no change in the cumulative release was observed afterward (up to 24 h). The 5-FU release behavior was similar between the two conditions evaluated; a slight increase in cumulative release at pH 5 and 40 °C was observed only for the case of NG-NVCL. In these conditions, pH does not influence the release; the relevant parameter is the temperature response of nanogels. However, the 3 °C increase in the system temperature, from 37 °C to 40 °C, can improve molecular diffusion in general, and a higher release rate was expected; this is perceptible with free 5-FU, with an increase of around 5% at 40 °C compared with 37 °C. Overall, the release percentage of 5-FU from NGs was lower than that of the free drug, demonstrating that the nanocarrier modulates the release rate. The release percentages of 5-FU were higher for NG-NVCL and NG-NVCL-VP with 80 and 60%, respectively, after two hours, as compared to the NGs containing LAMA. Likewise, NG-NVCL released the highest percentage of 5-FU since its VPTT is 32 °C for the unloaded system; therefore, it collapsed rapidly at the temperature of the study. For NGs containing LAMA, NG-NVCL-L (VPTT = 36 °C) and NG-NVCL-L-VP (VPTT = 39 °C) achieved only around 50% and 40% of 5-FU release, respectively, after two hours, and there was no further release after 24 h (Figure 8b). These NGs have more sites available to establish hydrogen bonds with 5-FU, strongly retaining the drug even at 40 °C (temperature higher than its VPTT). Furthermore, it is possible that the VPTT was modified due to the interactions of the nanogels with the loaded compounds.

In contrast, the CUR release profiles are much slower in comparison to 5-FU. All NGs released approximately 25% of CUR under physiological conditions (pH 7.4, 37 °C) but after 140 h (Figure 8c). An enhanced release of around 50% was achieved under slightly acidic conditions (pH 5) and 40 °C but also in a slow fashion as shown in Figure 8d. This could be beneficial, as it suggests CUR is released to a greater extent inside the cells. Further, it is observed that the cumulative CUR release from NG-NVCL and NG-NVCL-VP was superior compared to NGs containing LAMA. Both nanogels have a larger core and size compared to NGs with LAMA; therefore, the effect of acidic environment will have a larger effect, better expelling the loaded drug. Overall, the nanocarriers loaded with 5-FU and CUR will release fast 5-FU (2 h) for a “burst” chemotherapeutic effect, while CUR is released slowly, increasing the therapeutic effect even after 144 h, and the release is accelerated in endosomal conditions. The combination of the two drugs inside one nanocarrier has the potential for a more effective therapeutic outcome. The thermal effect is not as big as expected, in part probably because of the interactions of the nanogel with the loadings, while the pH effect is only important for CUR release.

### 2.5. Effect of NIR-Irradiation on 5-FU and CUR Release from Nanogels

**Figure 9:**
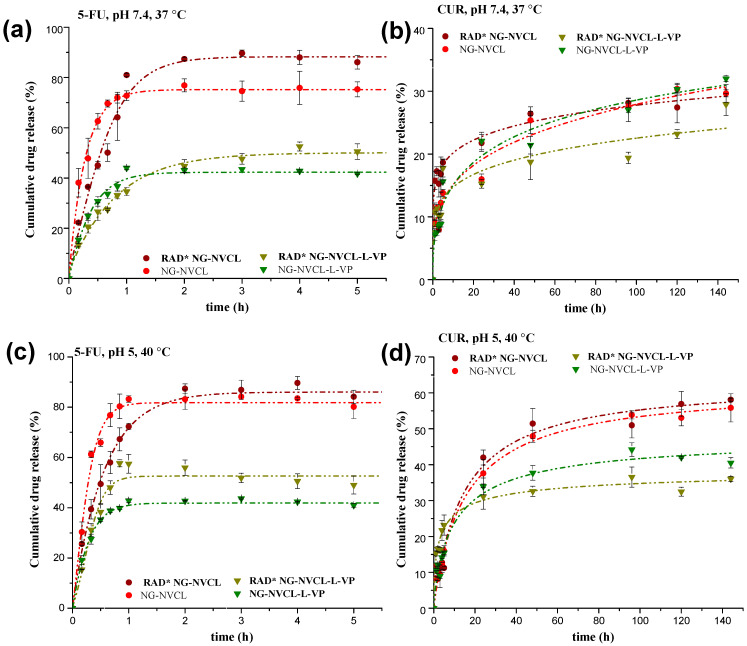
Release profiles of 5-FU and CUR at (**a**,**b**) pH 7.4, 37 °C, and (**c**,**d**) pH 5, 40 °C, through NG-NVCL and NG-NVCL-L-VP with/without NIR irradiation.

Release studies were carried out for NGs loaded with 5-FU, CUR, and GNRDs, subjected to intermittent NIR laser irradiation. Comparison of release profiles of NGs individually with/without NIR are shown in Figure S17 and Figure S18 for 5-FU and CUR, respectively. Figure 9 exemplifies selected release profiles from NGs (NG-NVCL and NG-NVCL-L-VP) to compare systems with/without LAMA and with/without NIR irradiation. Release profiles exhibited by 5-FU and CUR under the study conditions are described; at NIR irradiation, localized heating by the GNRDs is expected to increase NGs temperature above the experiment temperature by 3 °C, surpassing its VPTT. This shall result in nanogel network structure contraction and accelerated drug release if the drug–nanogel interactions are weak. This is the case for 5-FU; for all irradiated systems, a faster release of 5-FU compared to the non-irradiated systems is observed. The 5-FU release rate increased by approximately 10% at both experimental conditions tested, except for NG-NVCL at pH 5 and 40 °C, where around a 5% higher release rate was observed (Figure 9c). For this specific NG, at 40 °C, the NG-NVCL is almost totally shrunken (VPTT of 32 °C), so the NIR effect plays only a minor role. Under pH 5 and 40 °C conditions with NIR irradiation, the cumulative 5-FU release from NGs was (after 2.5 h): 87% for NG-NVCL (Figure 9c), 78% for NG-NVCL-VP (Figure S17f), and 62% for NG-NVCL-L (Figure S17g), and NG-NVCL-L-VP released up to 53% of 5-FU (Figure 9c), confirming the comparative behavior of NGs containing LAMA to those without LAMA already discussed without irradiation. The increase in 5-FU release with NIR irradiation has been reported previously using other thermoresponsive nanocarriers such as PNIPAAM nanogels with gold nanoparticles [60].

CUR release from NGs irradiated with NIR shows an opposite behavior to 5-FU release at long times; the CUR release rate decreased slightly as compared to non-irradiated systems (Figure 9b,d). However, at longer release times, the cumulative release without irradiation is larger than with irradiation. As shown in Figure S19, only the CUR system (without NG) has not exhibited a significant change in release behavior with concomitant irradiation, particularly at pH 7.4 and 37 °C. Likewise, in the case of NG-NVCL, the release profiles were similar at both experiment conditions, so the NIR irradiation effect was low. However, in the case of LAMA and NVP containing NGs, the detrimental effect of NIR irradiation on the cumulative release is clearly larger. CUR is located in the core of NGs and is released slowly since it is a hydrophobic molecule; NIR irradiation causes localized heating in NGs with GNRDs, and the sudden changes in the polymer structure could restrict CUR passage through the network, slowing the diffusion process. Ensuring the release of CUR is complex because, as the VPTT occurs and hydrophobic interactions in PNVCL increase, CUR can be trapped or preferred to remain inside the core due to increased hydrophobic interactions. In any case, the release rate of CUR is mainly affected by the pH of the environment, as discussed in the experiments without irradiation. Overall, and taking NG-NVCL as an example (pH 5 and 40 °C), in irradiation conditions, 5-FU releases up to 80% of the encapsulated drug in 24 h, while CUR release would be slower, reaching 40% at 24 h, but more CUR is released in the following 4 days, reaching 55% of the encapsulated compound. This behavior for CUR release may be favorable since a controlled release is desirable, prolonging its effect to the days following 5-FU treatment and possibly helping to reduce adverse symptoms caused by chemotherapy.

### 2.6. Models and Kinetic Parameters of Release Behavior of 5-FU and CUR from Nanogels With/Without NIR Irradiation

Kinetic parameters were determined to analyze the rate and mechanism release of 5-FU and CUR from the polymeric nanocarrier matrices, fitting the experimental release data to mathematical models with the following Equations (1)–(3):

First order: *F =* 1 − *e^−kt^* (1)

Higuchi equation: *F = kt^−^*^0.5^ (2)

Peppas equation: *F = k*^n^ (3)

where *F* is the fractional drug release, *k* is the release rate constant, and n is the diffusional exponent.

The effect of NIR irradiation on the release rate and the release mechanism of 5-FU and CUR was studied by adjusting the release data to kinetic models. A comparison was made between irradiated and non-irradiated conditions for 5-FU and CUR. The results obtained from the fit to the models of release profiles with/without NIR irradiation at conditions of pH 7.4 and 37 °C are summarized in Table S1 and Table S2, for 5-FU and CUR, respectively. The data from simulated conditions of endosomal pH and slight hyperthermia (40 °C) are shown in Table 4 for 5-FU and Table 5 for CUR. The criterion used for the best fit was based on the value of the linear correlation coefficient (r^2^).

Generally, for non-irradiated systems, Higuchi and Peppas models provided the best fit for 5-FU release under the experimental conditions evaluated. The Higuchi model is based on Fick’s law of diffusion. This result is consistent with previous studies [19,61]. On the other hand, the Peppas model provided the best fit for CUR release behavior. In the context of a core–shell nanogels, CUR molecules inside the core gradually diffuse through the polymer matrix. The value of *n* obtained in the Peppas model provides information on the release mechanism as dominated by diffusion, polymer relaxation, or the geometry of the polymer matrix. All values for *n* reported for both 5-FU and CUR are values of n ≤ 0.45. This suggests a Fickian-type release, which is associated with a diffusion-controlled process, accelerated by the contraction of the networks in response to temperature in the case of 5-FU [62]. Overall, the rate constant values in most models are superior for systems at pH 5 and 40 °C, reflecting the faster release in the combined pH and temperature effect. For NIR irradiated systems, the Higuchi and Peppas models provided the best fit under experimental conditions for both 5-FU and CUR. All values of *n* reported for CUR show values of n ≤ 0.45; this suggests a Fickian release; the diffusion mechanism was similar to non-irradiated conditions, and the rate constant values were lower in all models for NGs releasing CUR. In most NGs, release of 5-FU under irradiated conditions exhibited n values in the range 0.45 < n < 1.0, which is associated with an anomalous diffusion behavior termed non-Fickian diffusion [62].

The n values are oriented toward the limit of 0.45; the release would be influenced by diffusion as well as other additional processes; for example, structural changes in the nanogel that may have been triggered by NIR irradiation, affecting the release mechanism. Variations in the release mechanism have also been observed in other NIR-irradiated systems for DOX [63]. Furthermore, it was observed that the values for *k* were higher for the irradiated systems releasing 5-FU from most nanogels, except for NG-NVCL, where it remains similar. In the case of CUR as already discussed, the release rate is slower after NIR irradiation; therefore, the values for *k* were lower except for NG-NVCL.

### 2.7. In Vitro Chemo and Chemo-Photo Thermal Treatments

**Figure 10:**
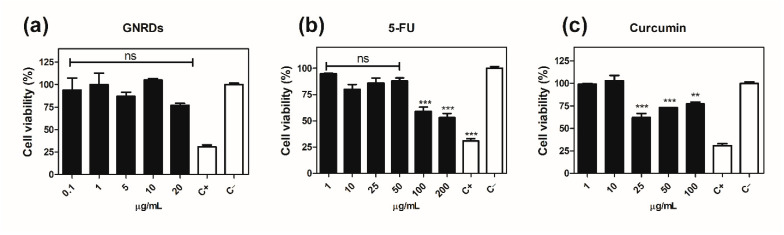
Quantitative evaluation of cell viability for HCT-116 cells treated with free drugs and GNRDs at 24 h: (**a**) GNRDs; (**b**) 5-FU drug; (**c**) curcumin. C+ (DMSO 10%), C− (PBS). ANOVA test, ** *p* < 0.001, *** *p* < 0.0001 and ns: no significant difference compared to the control C−.

**Figure 11:**
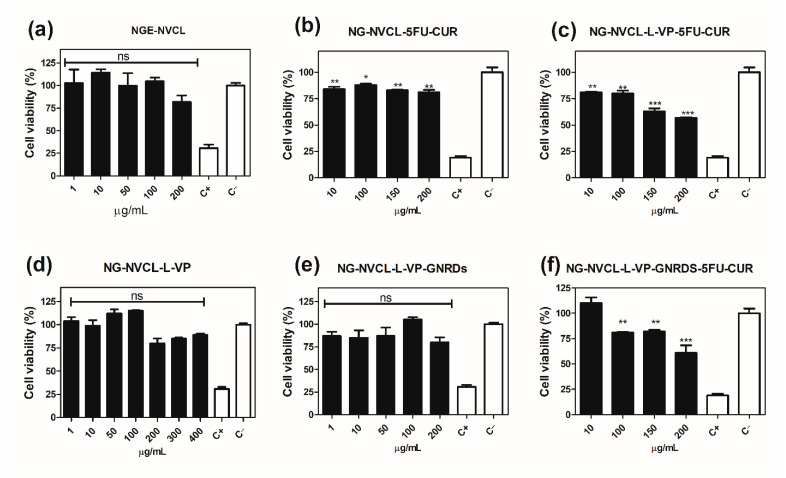
Quantitative evaluation of cell viability for HCT-116 cells treated with empty and loaded nanogels at 24 h: (**a**) NG-NVCL, (**b**) NG-NVCL-5FU-CUR, (**c**) NG-NVCL-L-VP-5FU-CUR, (**d**) NG-NVCL-L-VP, (**e**) NG-NVCL-L-VP-GNRDs, (**f**) NG-NVCL-L-VP-GNRDs-5FU-CUR, C+ (DMSO 10%), C− (PBS). ANOVA test, * *p* < 0.01, ** *p* < 0.001, *** *p* < 0.0001 and ns: no significant difference compared to the control C−.

**Figure 12:**
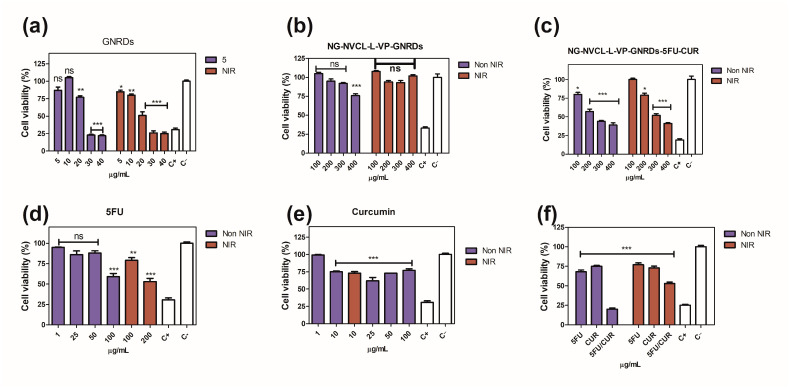
Comparative in vitro study of cell viability for HCT-116 cells treated with empty and loaded nanogels at 24 h, with NIR irradiation (5 min, 500 mW): (**a**) GNRDs; (**b**) NG-NVCL-L-VP-GNRDs; (**c**) NG-NVCL-L-VP-GNRDs-5FU-CUR; (**d**) 5-FU; (**e**) curcumin; (**f**) 5-FU (100 µg mL^−1^), CUR (20 µg mL^−1^), 5-FU/CUR (100/20 µg mL^−1^) combined, C+ (DMSO 10%), C− (PBS). ANOVA test, * *p* < 0.01, ** *p* < 0.001, *** *p* < 0.0001 and ns: no significant difference compared to the control C−.

The photo-thermal behavior of the drugs, GNRDs, and loaded nanogels in an aqueous medium was investigated with a 785 nm NIR laser. HCT-116 cells demonstrated different sensitivity to drugs and GNRDs without laser radiation (Figure 10). After 24 h incubation, it is observed that, as its concentration increases, cell viability decreases. The used drugs (5-FU and CUR) show a decrease in cell viability, which makes them useful in this type of cancer cell line. In the case of loaded nanogels, they show a cytotoxic effect only after 200 µg mL^−1^ due to the low drug load (Figure 11); even so, it was a significant effect by chemo-treatments. The unloaded nanogels were found to be non-cytotoxic up to a concentration of 200 µg mL^−1^ (Figure 11a,d). One of the important aspects to evaluate was the synergy of the effect of the drugs and NIR irradiation. Although the cells had been treated with unloaded nanogels, they withstood NIR irradiation, maintaining their levels of viable cells without significant changes (Figure 12b). This suggests that the nanogels are indeed biocompatible. The effect of irradiation was expected in the nanogels that contained GNRDs, as has been reported in the literature. However, the results showed that cells were resistant to irradiation; in fact, a slight increase in cell viability was observed, which was an interesting result (Figure 12). Cell proliferation is a very important physiological effect for the low-power laser irradiation (LPLI) used in clinical practice. Increased proliferation after LPLI has been shown in many cell types in vitro. Red to near-infrared light is thought to be absorbed by mitochondrial respiratory chain components, resulting in an increase in Reactive Oxygen Species (ROS), and adenosine triphosphate (ATP)/or cyclic AMP, and initiating a signaling cascade, which promotes cellular proliferation and cytoprotection. Following increased ATP and protein synthesis after LPLI, the expressions of growth factors and cytokines increase and ultimately lead to cell proliferation [64].

Initially, 200 µg mL^−1^ of loaded nanogels were used, then we doubled this concentration to 400 µg mL^−1^ to have twice the amount of GNRDs, and the effect from the irradiation was not notable (Figure 12b,c). Although the drug and GNRD-loaded nanogels decrease the cell viability with/without NIR irradiation to desired levels, e.g., 50% (Figure 12c), these results suggest that a cytotoxic effect from irradiation will not be obtained in all cases; it may depend on the resistance of the cell line. Resistance can arise from factors like the cell’s ability to tolerate heat and the amount of light absorbed by the nanoparticles, and there could be factors causing the aggregation of gold nanorods. The biocompatibility and structural stability of GNRDs directly determine their photothermal conversion efficiency and potential for applications in medical biology. GNRD synthesis schemes and purification processes can affect the nanorod aspect ratio, CTAB content, surface coating, surface charge, and cell culture medium stability, regulating cellular uptake processes and cell viability, ultimately determining the nanorod–cell interaction [65].

Material design and formulations for integrated drug delivery of anticancer drugs and NIR-responsive material are highlighted against the background of their potential capacity in optimizing the efficacy of cancer treatment. Despite the tremendous attention and encouraging results on GNRDs, some important issues should be addressed before further applications. For example, GNRDs could be deformed upon high-power laser irradiation, leading to loss of LSPR absorption in the NIR region [66].

## 3. Conclusions

Nanogels based on poly(*N*-vinylcaprolactam-*co*-*N*-vinylpyrrolidone) (P(NVCL-co-NVP)) with a poly(ethylene glycol) methyl ether methacrylate (PEGMA) shell and galactose (GAL) targeting ligand for colon cancer therapy were synthesized by SFEP. The sizes obtained for nanogels containing 5-FU, CUR, and GNRDs were between 126 and 320 nm. The VPTT at pH 7.4 was between 32 and 39 °C.

Furthermore, nanogels were able to encapsulate 5-FU and CUR with drug loading up to 35% and 3%, respectively. Likewise, the percentage of gold (%Au) was ≤ 10% for all NGs. On the one hand, the 5-FU release behavior was similar between the two conditions evaluated (pH 7.4/37 °C and pH 5/40 °C) without irradiation. Nevertheless, NIR irradiation significantly enhances the release rate of 5-FU from nanogels, especially in systems where drug–nanogel interactions are weak and the nanogel’s VPTT is exceeded. On the other hand, the CUR release profile from nanogels at pH 5 and 40 °C was higher than the release profile at pH 7.4 and 37 °C without irradiation. However, CUR release from NGs irradiated with NIR shows an opposite behavior to 5-FU release at long times; CUR release rate decreased slightly as compared to non-irradiated systems. This behavior for CUR release may be favorable since a controlled release is desirable, prolonging its effect to the days following 5-FU treatment, possibly helping to reduce adverse symptoms caused by chemotherapy.

The photothermal behavior and cytotoxicity of nanogels loaded with drugs and gold nanorods (GNRDs) were evaluated in HCT-116 colorectal cancer cells. In vitro evaluations indicated that the LAMA-functionalized PNVCL empty nanogels are biocompatible and do not present significant cytotoxicity up to concentrations of 200 μg mL^−1^. Moreover, the cytotoxic effect of 5-FU and CUR was preserved after loading in the nanogels, and the dosage for IC_50_ was achieved at 300 μg mL^−1^. NIR irradiation (5 min, 500 mW, single dose) did not induce a further reduction in cell viability in HCT-116 cells, probably because the concentration of GNRDs was not high enough to produce hyperthermia, although a resistance effect cannot be ruled out. These results demonstrate the complexity of nanomaterials-based chemo-photothermal combination therapy and the importance of research on the topic. Further studies are required to gain a deeper understanding of cellular resistance mechanisms and to refine integrated drug and NIR-responsive nanomaterial delivery systems for cancer treatment.

## 4. Materials and Methods

### 4.1. Materials

N-vinylcaprolactam (NVCL, 98%, Sigma-Aldrich Toluca, Mexico) was purified by recrystallization in hexane and dried under vacuum before use. N-vinylpyrrolidone (NVP, 99%, Sigma-Aldrich Toluca, Mexico) was purified by distillation. Poly(ethylene glycol) methyl ether methacrylate (PEGMA, M_n_ = 950 g mol^−1^, Sigma-Aldrich, Toluca, Mexico) was used as received, and ethylene glycol dimethacrylate (EGDMA, 98%, Sigma-Aldrich, Toluca, Mexico) was purified by filtration through an inhibitor remover column for hydroquinones (Sigma-Aldrich, Toluca, Mexico). The GAL-functionalized monomer 2-lactobionamidoethyl methacrylate (LAMA) was synthesized following a previously reported procedure [37]. 5-Fluorouracil (5-FU, 98%, Sigma-Aldrich Toluca, Mexico) and curcumin (CUR, 97%, TCI, Tokyo, Japan) were used as received. Potassium persulfate (KPS, 98%), cetyltrimethylammonium bromide (CTAB, 99%), gold (III) chloride hydrate, sodium borohydride (NaBH_4_, 99%), silver nitrate (AgNO_3_, 99%), sulfuric acid (H_2_SO_4_, 98%), ascorbic acid (AA, 98%), deuterated chloroform (CDCl_3_, 99.8%), deuterated dimethyl sulfoxide (DMSO-d_6_, 99%), sodium chloride (99.4%), dibasic sodium phosphate anhydrous (99%), monobasic potassium phosphate (98%), and tween 80 (Polysorbate 80) were all used as received from Sigma-Aldrich (Toluca, Mexico). Phosphate buffer solutions were prepared at 0.05 M using commercial-grade distilled water (Parents Choice, CA, USA).

Cell line HCT-116 (CCL-247) was obtained from ATCC (Manassas, VA, USA), McCoy 5a media was obtained from Corning (New York, NY, USA), and cell culture antibiotic/antimycotic solution was obtained from Sigma-Aldrich. CellTiter 96^®^ Aqueous One Solution Cell Proliferation Assay (MTS) was purchased from Promega (Madison, WI, USA).

### 4.2. Synthesis of Thermoresponsive and Galacto-Functionalized NGs

NGs were synthesized by surfactant-free emulsion polymerization (SFEP) through a free-radical mechanism. The methodology was adapted from the report of Gonzalez-Ayon et al. [67]. A series of PNVCL-PEGMA NGs were prepared by adding different monomers, such as NVP and/or LAMA, and using PEGMA as a stabilizer. EGDMA and KPS were used as crosslinker and thermal initiator, respectively. The synthesis of one nanogel is described in detail (NG-NVCL-L-VP): 350 mL of distilled water was placed in a 500 mL jacketed batch reactor (Syrris, model Atlas Potassium, Royston, UK) equipped with an automated system, connected to a nitrogen inlet. The temperature was adjusted to 80 °C with constant stirring at 400 rpm. Subsequently, 2.16 g of NVCL (15.52 mmol), NVP (184 µL, 1.73 mmol), LAMA (0.216 g, 0.46 mmol), 1.6 g (1.68 mmol) of PEGMA, and crosslinker EGDMA (114 µL, 0.074 mmol) were added. The mixture was then stirred for 20 min. Afterward, 0.0187 g (0.69 mmol) of KPS was dissolved in 10 mL of distilled water and then injected into the reaction system to begin the polymerization; the reaction was stopped after 30 min by cooling. The resulting products were purified via dialysis using a Spectra/Por^®^ dialysis membrane (MWCO = 12–14 kDa) (Repligen, Waltham, MA, USA) against deionized water for 5 days, with water changes performed once daily. Finally, the samples were frozen and lyophilized using a Labconco´s Freeze Dry System model Freezone 4.5 (Kansas City, MI, USA). The dried samples were stored until their characterization.

### 4.3. Preparation of GNRDs

GNRDs were synthesized in an aqueous CTAB solution using a seed growth method as previously reported procedure [67]. Gold seeds were obtained by mixing HAuCl_4_ (10 mM, 0.25 mL) with CTAB (0.1 M, 7.5 mL), followed by dropwise addition of a cold NaBH_4_ solution (10 mM, 600 µL) while stirring. The growth solution was prepared with HAuCl_4_ (10 mM, 5 mL), CTAB (0.1 M, 100 mL) solution, AgNO_3_ (10 mM, 1 mL), and ascorbic acid (100 mM, 0.8 mL). The seed solution (7.5 mL) was mixed with the growth solution (0.25 mL). GNRDs were purified through two cycles of centrifugation: first at 12,000× *g* rpm for 30 min, followed by another at 6000 rpm for 15 min, discarding the supernatant. The LSPR of the synthesized GNRDs was assured to be within the desired wavelength range (750–850 nm) by using UV-vis spectroscopy (Varian Cary 100, Agilent Technologies, Santa Clara, CA, USA).

### 4.4. Characterization of Nanogels

The composition of the NGs was determined using hydrogen nuclear magnetic resonance spectroscopy (1H-NMR) with a Bruker´s model Avance III (Billerica, MA, USA) spectrometer operating at 400 MHz. Dried samples of NGs (20 mg) were dissolved in deuterated chloroform (CDCl_3_) for the determination, and a minimum of 150 scans were collected.

The particle size distribution was assessed by Dynamic Light Scattering (DLS) using a Malvern Instruments Zetasizer Nano-ZS (ZEN 3690) (Malvern-Panalytical, Worcestershire, UK), with a red laser (630 nm) and a detector set at a 90° scattering angle, employing CONTIN analysis and the Stokes–Einstein equation for spherical particles. Furthermore, the temperature response of the NGs was evaluated by DLS also. The effect of temperature on particle size was assessed using dispersions of NGs at a concentration of 1 mg mL^−1^ in distilled water and PBS solutions at pH 7.4 and pH 5, running a heating program from 10 to 55/65 °C, respectively, allowing 3 min for an equilibration period at each temperature setting. The volume phase transition temperature (VPTT) was determined as the minimum of the derivative of dD_h_/dT. Additionally, the zeta potential (ζ) was measured using DLS equipment, which employs laser Doppler microelectrophoresis for this. These measurements were conducted at pH values between 3.5 and 9.5 in folded capillary cells at 25 °C.

The weight average molecular weight (M_w_), radius of gyration (R_g_), and second virial coefficient (A_2_) of NGs were determined using Static Light Scattering (SLS) with the Berry plot algorithm. Data were collected using a model BI-200SM-E multi-angle DLS/SLS goniometer from Brookhaven Instruments (Nashua, NH, USA). Nanogel dispersions were prepared at concentrations of 0.1, 0.15, 0.25, 0.35, and 0.5 mg mL^−1^ in filtered distilled water; the intensity of scattered light was measured across an angular range from 30° to 120°.

On the other hand, maximum of decomposition steps of the NGs (T_d_) and the gold content (Au%) in loaded NGs were obtained through Thermogravimetric Analysis (TGA) using a Simultaneous DSC-TGA, TA Instruments, model 2906 New Castle, DE, USA. The heating ramp was set at 10 °C min^−1^ from 20 to 600 °C with a nitrogen flow rate of 60 mL min^−1^.

### 4.5. Drug Loading and Encapsulation Efficiency

The loading of 5-FU and CUR was carried out simultaneously by the equilibrium swelling method. A total of 20 mg of lyophilized NGs was dispersed in 10 mL of an aqueous solution of 5-FU (2 mg mL^−1^), and subsequently, 250 µL of CUR solution in ethanol (10 mg mL^−1^) was added. The mixture obtained was stirred vigorously for 1 h, keeping the dispersion cold (ice bath). Afterward, the mixture was stirred for 24 h at room temperature and then refrigerated for 24 h at 4 °C. Finally, the mixture was centrifuged at 3000× *g* rpm for 5 min to remove insoluble CUR. The supernatant was carefully collected, and loaded NGs were dialyzed against water for 3 h at room temperature using a Spectra/Por^®^ Dialysis Membrane MWCO, 12–14 kDa, and changing the water every 0.5 h to remove non-encapsulated 5-FU. The loaded NGs were then frozen in a conventional freezer for 12 h and lyophilized. Subsequently, loaded NGs were analyzed by UV-visible spectroscopy to determine the drug content at wavelengths of 266 nm for 5-FU and 428 nm for CUR. The drug-loaded content (*DLC*) and loading efficiency (*DLE*) were determined using Equations (4) and (5), respectively.

(4) $$DLC\;\left(\%\right)=\frac{W_{LD}}{W_{NG}+W_{LD}}\;*100\%$$

(5) $$DLE\;\left(\%\right)=\frac{W_{LD}}{W_{FD}}*100\%$$

where *W_LD_* is the mass of 5-FU or CUR loaded in the NGs, *W_NG_* is the mass of NGs, and *W_FD_* is the mass of 5-FU or CUR feed.

### 4.6. GNRDs Loading into NGs

To load GNRDs into the nanogel, 1 mL of a GNRD solution (2.5 µg mL^−1^ in deionized water) was added to 10 mL of a 5-FU-CUR-loaded nanogel solution and stirred at 100 rpm for 1 h. Then, the mixture was incubated at 4 °C for 48 h. Unstable GNRDs tend to precipitate after incubation, so only the supernatant (GNRDs loaded in NGs) was retained. The NGs with GNRDs were subsequently frozen in a conventional freezer for 12 h and lyophilized using a Labconco´s Freeze Dry System model Freezone 4.5 (Kansas City, MI, USA). The percentage of GNRDs loaded into the NGs was approximated by the weight difference in residue, determined by TGA, and the percentage of gold (%*Au*) was calculated by Equation (6).

(6) $$\%Au=\frac{R_{LNG}-R_{NG}}{W_{LNG}}*100\%$$

where *R_LNG_* is the mass of residue of loaded nanogel, *R_NG_* is the mass of residue of nanogel (obtained by TGA at 550 °C), and *W_LNG_* is the mass of loaded nanogel.

### 4.7. In Vitro Release Studies of 5-FU and CUR With/Without NIR Irradiation

In vitro release experiments were performed under sink conditions with controlled temperature, pH, and agitation. Two conditions were simulated: on the one hand, mimicking the environment of healthy cells (37 °C and pH 7.4) and, on the other hand, the hyperthermia and endosomal environment in cancer cells (40 °C and pH 5). For a typical experiment, 5 mg of loaded NGs was dispersed in 3 mL of PBS buffer solution and placed into a dialysis membrane (Spectra/Por^®^ Dialysis Membrane, MWCO: 12–14 kDa) and then was immersed in 90 mL of release medium containing 0.5% *v*/*v* of Tween 80 in PBS buffer, stirred at 70 rpm at a specified temperature and pH. At defined time intervals, an aliquot (3 mL) was withdrawn, and an equal volume of fresh medium was subsequently added to maintain a constant volume.

For release experiments using NIR irradiation, NGs loaded with 5-FU, CUR, and GNRDs (GNRDs-5-FU-CUR-loaded nanogel) were used. The loaded NGs were exposed to intermittent irradiation with a near-infrared laser (Micro Laser Systems Inc., Fermion III) at 808 nm and an output power of 500 mW located at 10 cm above the dialysis bag, irradiating for 5 min every hour during the first 5 h. The following measurements (one per day) were taken, and the samples were irradiated for 5 min before taking an aliquot. Drug release experiments were performed until 24 h and 144 h for 5-FU and CUR, respectively. Cumulative drug releases for 5-FU and CUR (%DR) were determined by UV-vis spectroscopy and calculated by Equation (7) using a standard calibration curve measured at 266 and 428 nm for 5-FU and CUR, respectively. Measurements were performed in triplicate; the average and standard deviation are reported.

(7) $$\%DR=\frac{W_{DR}}{W_{DL}}*100\%$$

*W_DR_* is the mass of drug released from NGs, and *W_DL_* is the mass of total drug loaded into NGs.

### 4.8. Cell Viability Assay

Human colon cancer cells (HCT-116) were cultured in McCoy 5a medium supplemented with 10% FBS and 1% antibiotic/antimycotic solution in a humidified atmosphere with 5% CO_2_ at 37 °C. Cells were seeded into a 96-well plate at a density of 5000 cell/well and incubated for 24 h. Then, the media was replaced with different treatments (diluted in fresh media) and incubated for another 24 h. The treatments that were evaluated were empty nanogels (NG-NVCL, NG-NVCL-L-VP, 1–200 µg mL^−1^), 5-FU (1–200 µg mL^−1^), CUR (1–100 µg mL^−1^), GNRDs (1–40 µg mL^−1^), nanogels (NG-NVCL-5-FU-CUR, NG-NVCL-L-VP-5-FU-CUR, 10–200 µg mL^−1^), and nanogels loaded with drugs and GNRDs (NG-NVCL-L-VP-GNRDs, NG-NVCL-L-VP-5-FU-CUR, 10–400 µg mL^−1^). Cell viability was evaluated with MTS. For this, the cells were washed with PBS, and a 10% MTS solution diluted in fresh media was added. Then, cells were incubated for 3 h. Finally, absorbance was measured at 492 nm.

### 4.9. Cell Viability Studies of Chemo/Photothermal Effect

Human colon cancer cells (HCT-116) were seeded into a 96-well plate at a density of 5000 cell/well and incubated for 24 h. Then, the media was replaced with different treatments (diluted in fresh media) and incubated for another 24 h. Subsequently, the cells were irradiated using a fiber-coupled diode laser (CNI laser, model MDL-III-785, Changchun, China) with a wavelength of 785 nm with a single NIR dose (output power 500 mW) for 5 min per well. They were incubated for 2 h before measuring cell viability using the MTS assay. All experiments were carried out in triplicate.

## Figures and Tables

**Table 1:** Composition by ^1^H-NMR and colloidal properties by DLS-SLS of NGs.

Nanogel		Product Composition by ^1^H-NMR (mol%)				Colloidal Properties				
	NVCL	NVP	LAMA	PEGMA	R_h_ ^(c)^ (nm)	PDI ^(c)^	M_w_ ^(d)^ (g mol^−1^))	R_g_ ^(d)^ (nm)	A_2_ ^(d)^ (cm^3^ mol g^2^)	Ρ ^(e)^
NG-NVCL ^(a)^	55	-	-	45	72 ± 29	0.29	1.06 × 10^7^	77 ± 4	2.56 × 10^−5^	1.06
NG-NVCL-VP ^(a)^	36	25	-	39	135 ± 63	0.26	1.46 × 10^8^	124 ± 20	1.83 × 10^−6^	0.92
NG-NVCL-L ^(b)^	49	-	15	36	37 ± 16	0.24	2.01 × 10^6^	51 ± 5	5.80 × 10^−5^	1.37
NG-NVCL-L-VP ^(b)^	35	22	11	32	43 ± 17	0.18	4.94 × 10^6^	58 ± 11	2.68 × 10^−5^	1.34

^(a)^ EGDMA 3 mol%; ^(b)^ EGDMA 3.5 mol%; ^(c)^ determined by DLS (R_h_ = D_h_/2); ^(d)^ determined by SLS; ^(e)^ ρ = R_g_/R_h_. Data of DLS and SLS were obtained in water at 25 °C.

**Table 2:** VPTT data of nanogels at different pHs.

Nanogel	VPTT (°C)		
	pH 5	pH 6.8	pH 7.4
NG-NVCL	45	34	32
NG-NVCL-VP	51	38	39
NG-NVCL-L	37	35	37
NG-NVCL-L-VP	39	39	39

**Table 3:** Size, zeta potential, and loading of 5-FU, CUR, and GNRDs in NGs.

Nanogel	D_h_ ^(a)^ (nm)	PDI ^(a)^	ζ ^(b)^ (mV)	5-FU ^(c)^		CUR ^(c)^		GNRDs
				*DLC* (wt%)	*DLE* (%)	*DLC* (wt%)	*DLE* (%)	%*Au* ^(d)^
NG-NVCL	223	0.394	−6	21	30	2	15	4
NG-NVCL-VP	320	0.455	−1.3	26	28	3	23	9
NG-NVCL-L	126	0.505	−4	32	37	2	14	10
NG-NVCL-L-VP	157	0.390	−11	35	40	2	15	7

^(a)^ Determined by DLS in deionized water at 25 °C; ^(b)^ measured at pH 7.4; ^(c)^ calculated by UV-vis spectroscopy based on the corresponding calibration curve; ^(d)^ determined by TGA.

**Table 4:** Comparison of kinetics parameters of the release of 5-FU from NGs with/without NIR irradiation at conditions of pH 5 and 40 °C.

Model	Nanogel	5-FU pH 5, 40 °C			
		Without NIR	NIR	Without NIR	NIR
		***k* (h^−1^)**		**r^2^**	
**First order**	NG-NVCL	0.8663	1.079	0.66	0.92
	NG-NVCL-VP	0.4692	0.5422	0.73	0.89
	NG-NVCL-L	0.3204	0.4544	0.59	0.77
	NG-NVCL-L-VP	0.2420	0.4182	0.59	0.64
		***k* (h^−0.5^)**		**r^2^**	
**Higuchi**	NG-NVCL	0.6460	0.655	0.83	0.98
	NG-NVCL-VP	0.4882	0.4903	0.89	0.95
	NG-NVCL-L	0.3966	0.4755	0.84	0.92
	NG-NVCL-L-VP	0.3250	0.4678	0.85	0.85
		***k* (h^−n^)**		**r^2^**	
**Peppas**	NG-NVCL	0.7977	0.6873	0.77	0.97
		n = 0.39	n = 0.51		
	NG-NVCL-VP	0.5688	0.5684	0.86	0.99
		n = 0.45	n = 0.33		
	NG-NVCL-L	0.4842	0.5356	0.82	0.90
		n = 0.39	n = 0.48		
	NG-NVCL-L-VP	0.4028	0.5250	0.86	0.83
		n = 0.34	n = 0.55		

**Table 5:** Comparison of kinetic parameters of the release of CUR from NGs with/without NIR irradiation at conditions of pH 5 and 40 °C.

Model	Nanogel	CUR pH 5, 40 °C			
		Without NIR	NIR	Without NIR	NIR
		***k* (h^−1^)**		**r^2^**	
**First order**	NG-NVCL	0.0056	0.0058	0.85	0.83
	NG-NVCL-VP	0.0052	0.0031	0.87	0.85
	NG-NVCL-L	0.0036	0.0022	0.69	0.56
	NG-NVCL-L-VP	0.0035	0.0021	0.75	0.74
		***k* (h^−0.5^)**		**r^2^**	
**Higuchi**	NG-NVCL	0.0487	0.0496	0.94	0.92
	NG-NVCL-VP	0.0440	0.0317	0.94	0.95
	NG-NVCL-L	0.0359	0.0243	0.86	0.75
	NG-NVCL-L-VP	0.0345	0.0223	0.89	0.75
		***k* (h^−n^)**		**r^2^**	
**Peppas**	NG-NVCL	0.0711	0.0829	0.96	0.92
		n = 0.44	n = 0.41		
	NG-NVCL-VP	0.1217	0.0687	0.95	0.97
		n = 0.31	n = 0.37		
	NG-NVCL-L	0.0947	0.1181	0.95	0.89
		n = 0.33	n = 0.24		
	NG-NVCL-L-VP	0.0913	0.1553	0.92	0.81
		n = 0.33	n = 0.18		

## Data Availability

The raw data supporting the conclusions of this article will be made available by the authors on request.

## Supplementary Materials

The following supporting information can be downloaded at: https://www.mdpi.com/article/10.3390/gels12010023/s1, Figure S1: ^1^H-NMR spectrum of nanogel NG-NVCL in CDCl_3_.; Figure S2: ^1^H-NMR spectrum of nanogel NG-NVCL-VP in CDCl_3_.; Figure S3: ^1^H-NMR spectrum of nanogel NG-NVCL-L in CDCl_3_.; Figure S4: ^1^H-NMR spectrum of nanogel NG-NVCL-L-VP in CDCl_3_.; Figure S5: Berry plot for nanogel NG-NVCL in water at 25 °C; Figure S6: Berry plot for nanogel NG-NVCL-VP in water at 25 °C; Figure S7: Berry plot for nanogel NG-NVCL-L in water at 25 °C; Figure S8: Berry plot for nanogel NG-NVCL-L-VP in water at 25 °C; Figure S9: Hydrodynamic diameter (D_h_) of NGs at 25 °C in: (a) deionized water, (b) PBS pH 7.4 and (c) PBS pH 5; Figure S10: Size analysis as a function of temperature by DLS at: pH 7.4 (a,d,g,j), pH 6.8 (b,e,h,k) and pH 5 (a,d,g,j) for NG-NVCL (a–c), NG-NVCL-VP (d–f), NG-NVCL-L (g–i) and NG-NVCL-L-VP (j–l); Figure S11: TEM Micrograph of NG-NVCL-L-VP loaded with GNRDs; Figure S12: Size distribution by DLS in PBS 7.4 before and after concurrent loading of 5-FU, CUR and GNRDs of NG-NVCL-VP (a) and NG-NVCL-L (b); Figure S13: TGA thermogram of unloaded NGs: (a) NG-NVCL, (b) NG-NVCL-VP, (c) NG-NVCL-L and (d) NG-NVCL-L-VP; Figure S14; TGA thermograms of loaded NGs: (a) NG-NVCL (5-FU + CUR + GNRDs), (b) NG-NVCL-VP (5-FU + CUR + GNRDs), (c) NG-NVCL-L (5-FU + CUR + GNRDs) and (d) NG-NVCL-L-VP (5-FU + CUR + GNRDs); Figure S15: Calibration curve and UV-Vis spectrum of CUR in PBS with Tween 80 (0.5%); Figure S16: Calibration curve and UV-Vis spectrum of 5-FU in PBS with Tween 80 (0.5%); Figure S17: Release profiles of 5-FU at pH 7.4, 37 °C (a–d) and at pH 5, 40 °C (e–h) from NGs with/without concomitant NIR irradiation; Figure S18: Release profiles of CUR at pH 7.4, 37 °C (a–d) and at pH 5, 40 °C (e–h) from NGs with/without concomitant NIR irradiation; Figure S19: Release profiles of free CUR at pH 7.4, 37 °C (a) and at pH 5, 40 °C (b) with/without concomitant NIR irradiation; Table S1: 5-FU in vitro release kinetics at pH 7.4, 37 °C: Data fitted with different mathematical models; Table S2: CUR in vitro release kinetics at pH 7.4, 37 °C: Data fitted with different mathematical models.
