# Supplementary material for: PNVCL-Based Multifunctional Nanogels Loaded with Curcumin, 5-Fluorouracil, and Gold Nanorods: Their Performance in Colon Cancer Cells
Source: Gels. 2025 Dec 25;12(1):23. doi: 10.3390/gels12010023 (PMC12841463; doi:10.3390/gels12010023)
Supplement: Supplementary file 1 [file gels-12-00023-s001.zip › gels-4047237-supplementary.pdf]

Supplementary Material

PNVCL-based multifunctional nanogels loaded with curcumin, 5-fluorouracil and gold nanorods: Their performance in colon cancer cells

Diana V. Félix-Alcalá[1], Mirian A. González-Ayón[1,]*, Lizbeth A. Manzanares-Guevara[2], Alexei F. Licea-Navarro[2], Eugenio R. Méndez[3], and Angel Licea-Claverie[1,]*

[1]Centro de Graduados e Investigación en Química, Tecnológico Nacional de México/Instituto Tecnológico de Tijuana, Apartado Postal 1166, Tijuana 22454, México.

[2]Departamento de Innovación Biomédica, Centro de Investigación Científica y de Educación Superior de Ensenada (CICESE), Ensenada 22860, B.C., México.

[3]Division de Física Aplicada, Centro de Investigación Científica y de Educación Superior de Ensenada (CICESE), Ensenada 22860, B.C., México.

* Correspondence: aliceac@tectijuana.mx (A.L.C.), mirian.gonzalez@tectijuana.edu.mx (M.A.G.-A.)

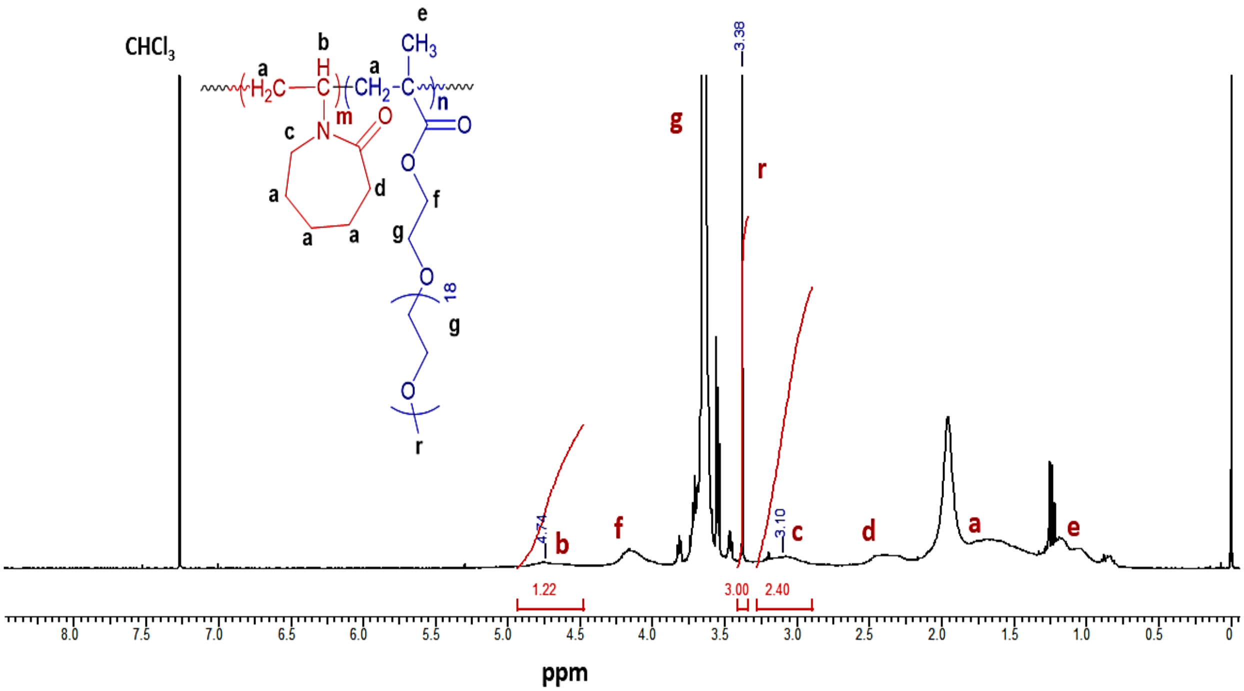

Figure S1. ^{1}H-NMR spectrum of nanogel NG-NVCL in $CDCl_3$.

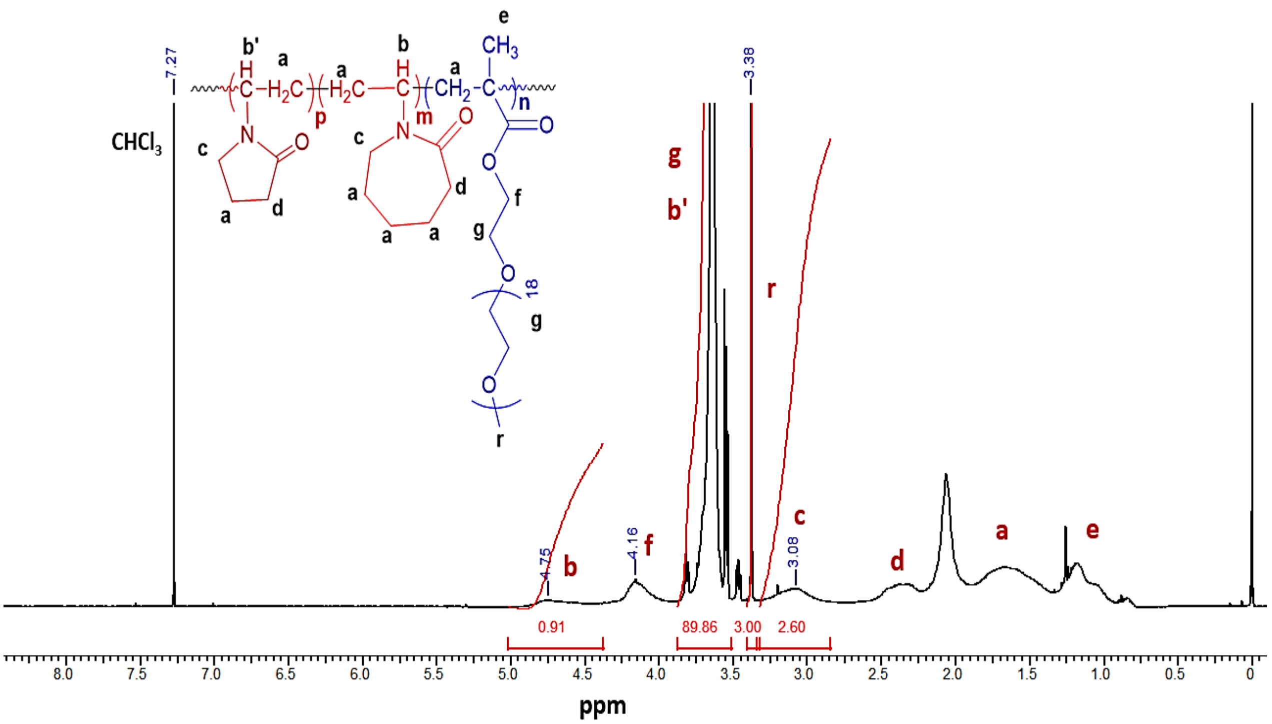

Figure S2. ^{1}H-NMR spectrum of nanogel NG-NVCL-VP in $CDCl_3$.

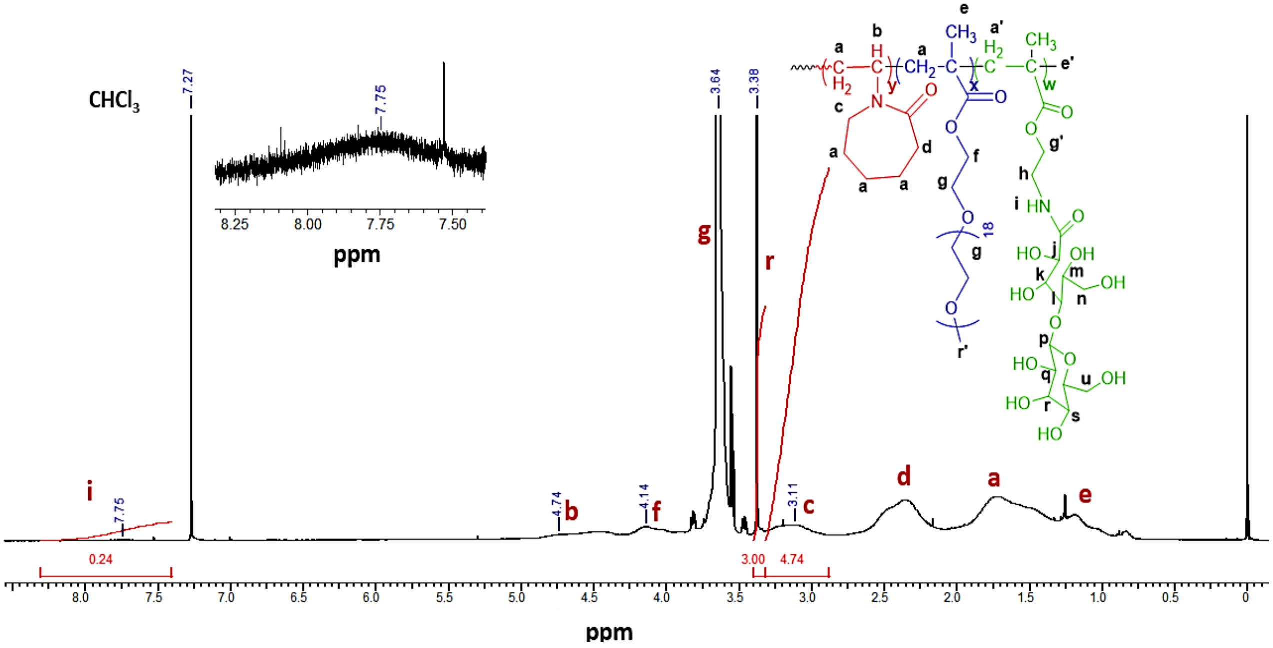

Figure S3. ^{1}H-NMR spectrum of nanogel NG-NVCL-L in $CDCl_3$.

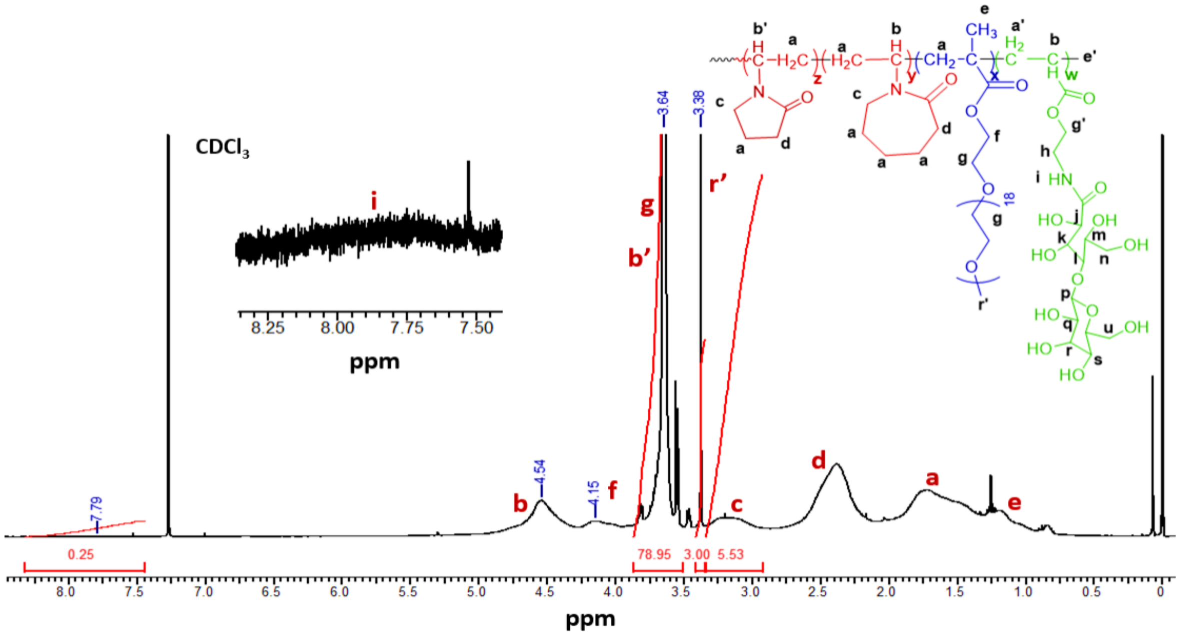

Figure S4. ^{1}H-NMR spectrum of nanogel NG-NVCL-L-VP in $CDCl_3$.

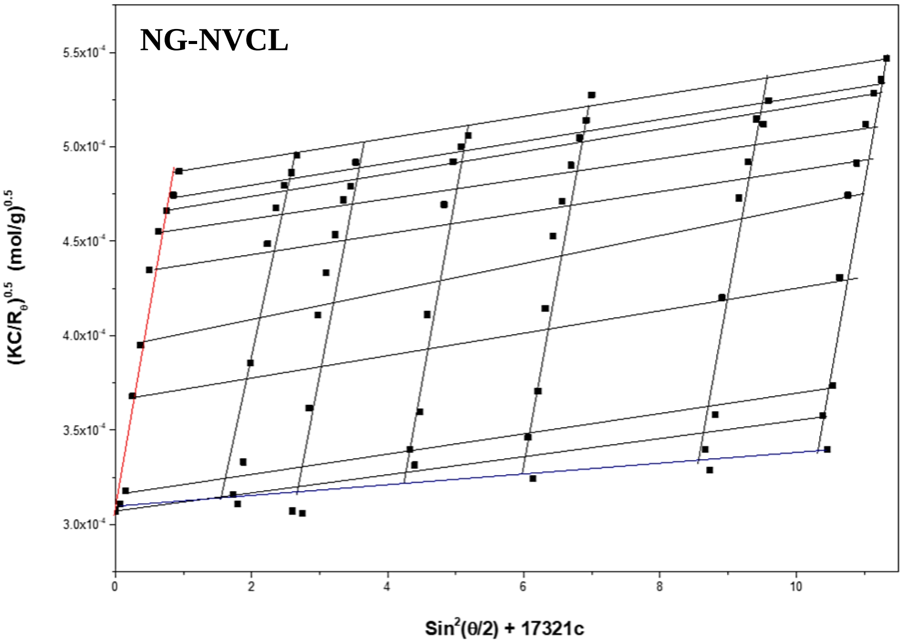

Figure S5. Berry plot for nanogel NG-NVCL in water at 25 °C.

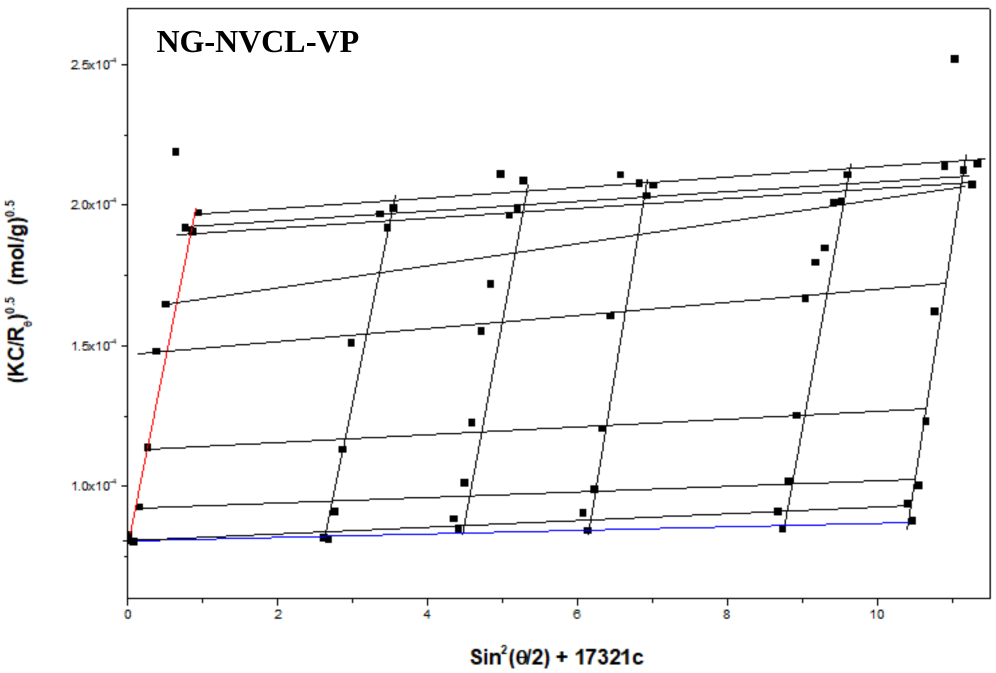

Figure S6. Berry plot for nanogel NG-NVCL-VP in water at 25 ^{o}C.

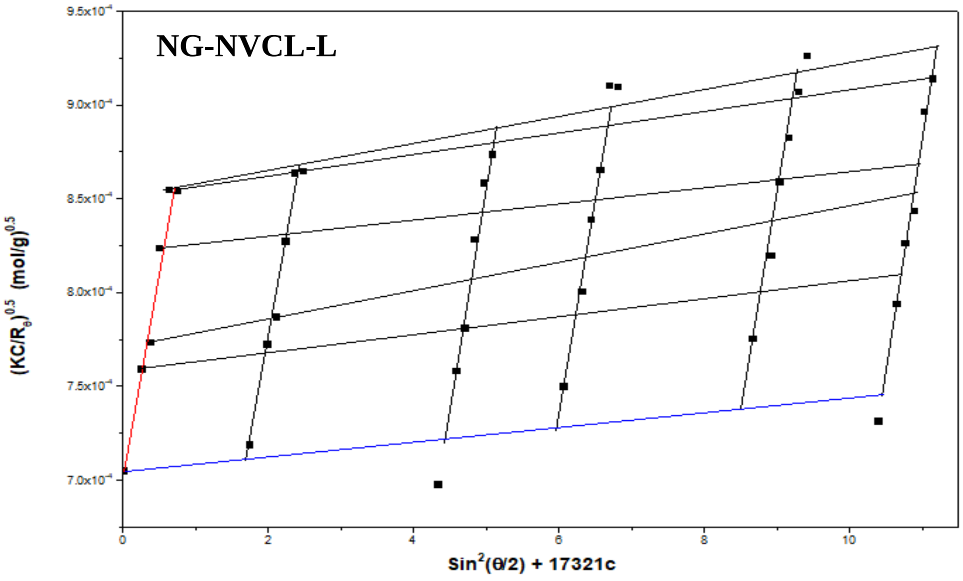

Figure S7. Berry plot for nanogel NG-NVCL-L in water at 25 ^{o}C.

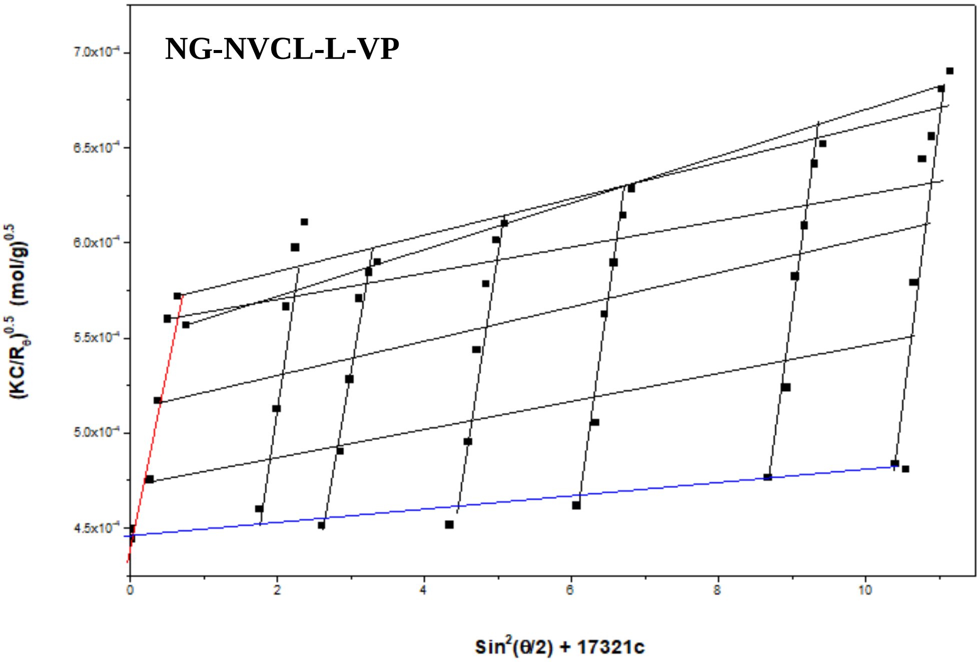

Figure S8. Berry plot for nanogel NG-NVCL-L-VP in water at 25 ^{o}C.

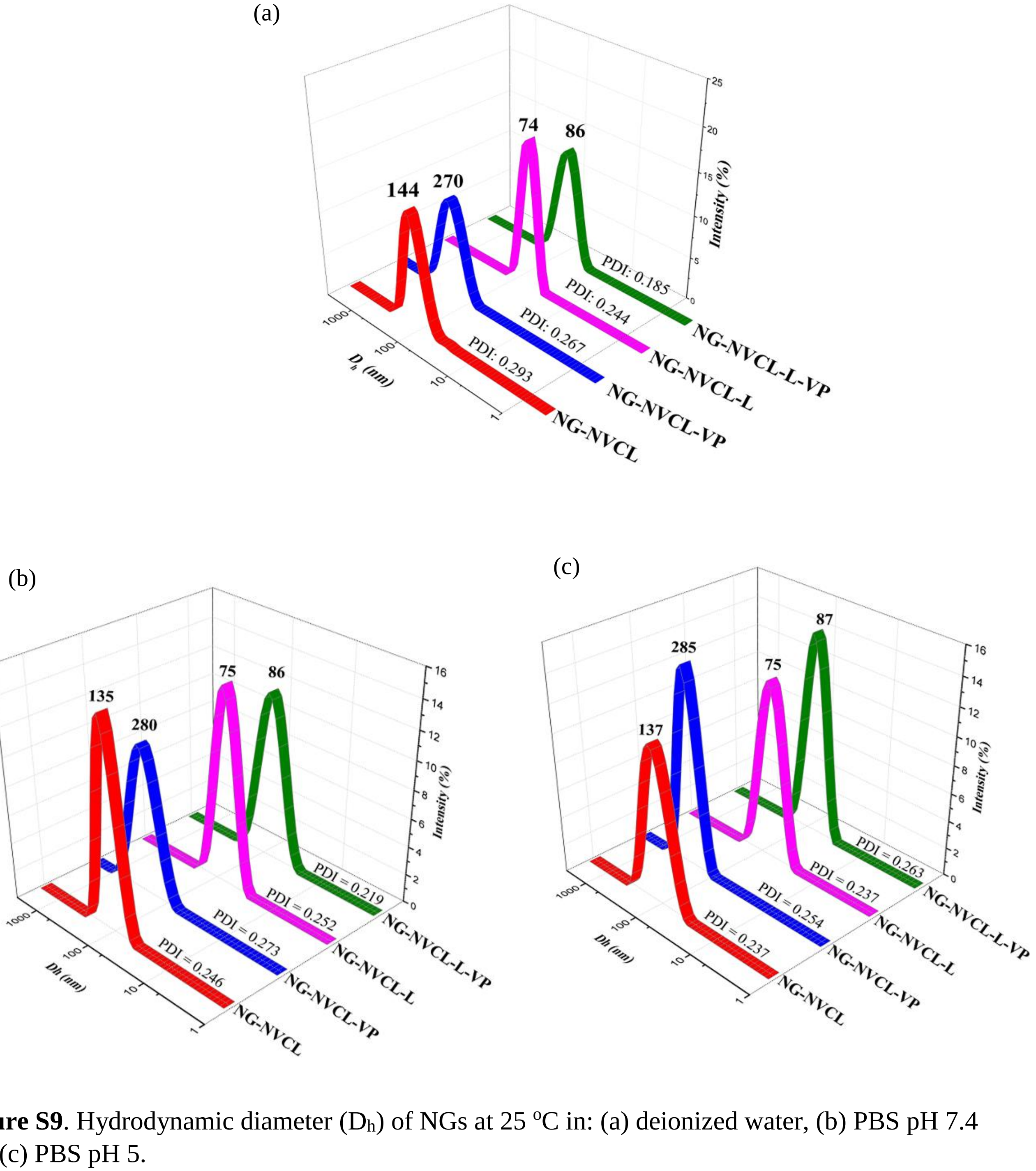

Figure S9. Hydrodynamic diameter (D_h) of NGs at 25 °C in: (a) deionized water, (b) PBS pH 7.4 and (c) PBS pH 5.

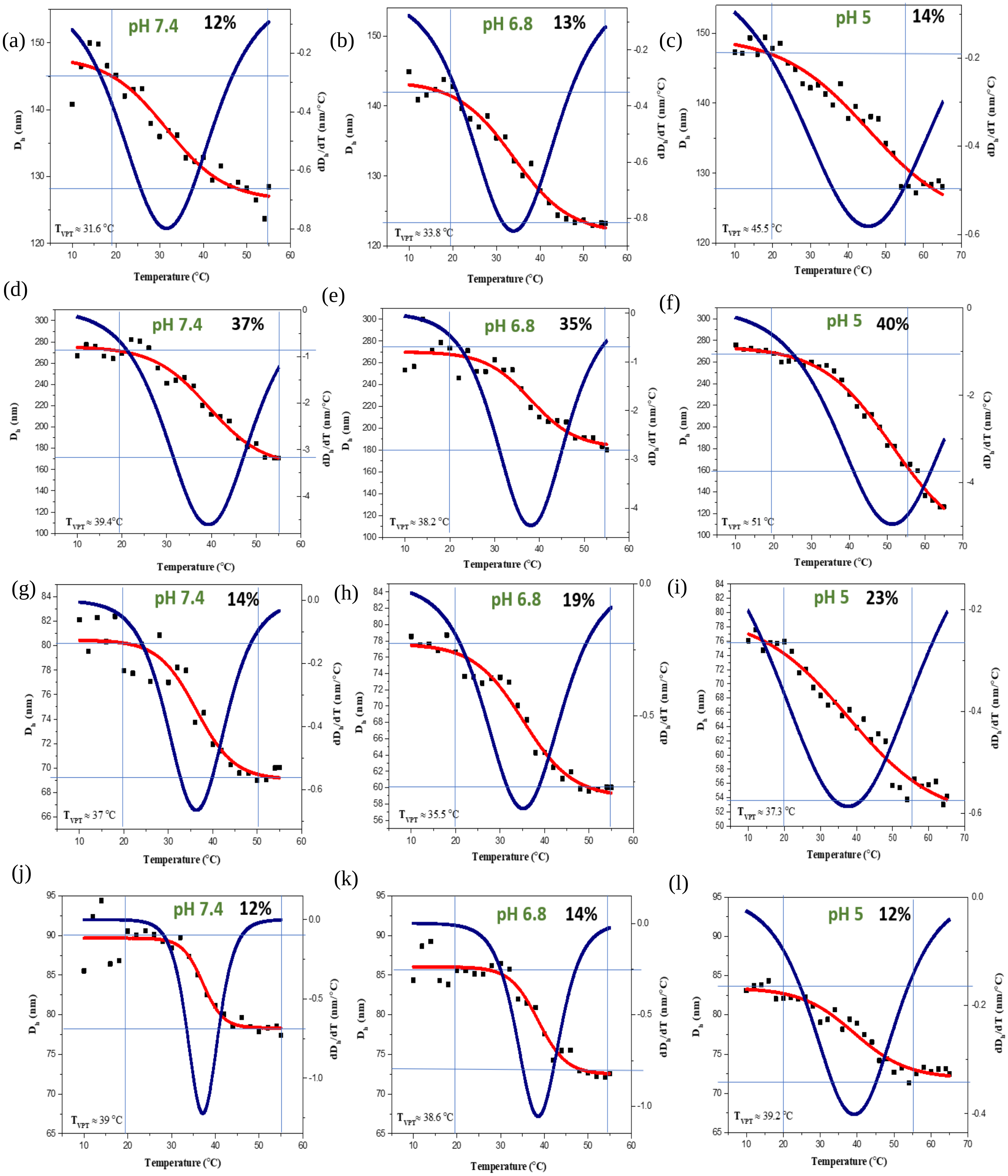

Figure S10. Size analysis as a function of temperature by DLS at: pH 7.4 (a.d,g,j) , pH 6.8 (b,e,h,k) and pH 5 (a.d,g,j) for NG-NVCL (a,b,c), NG-NVCL-VP (d,e,f), NG-NVCL-L (g,h,i) and NG-NVCL-L-VP (j,k,l).

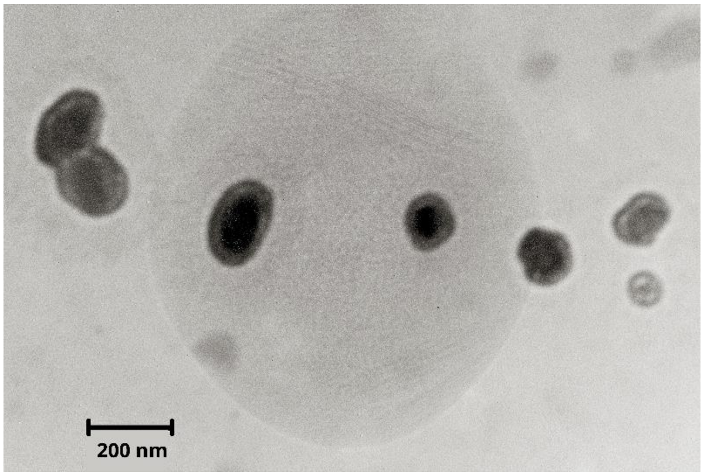

Figure S11. TEM Micrograph of NG-NVCL-L-VP loaded with GNRDs.

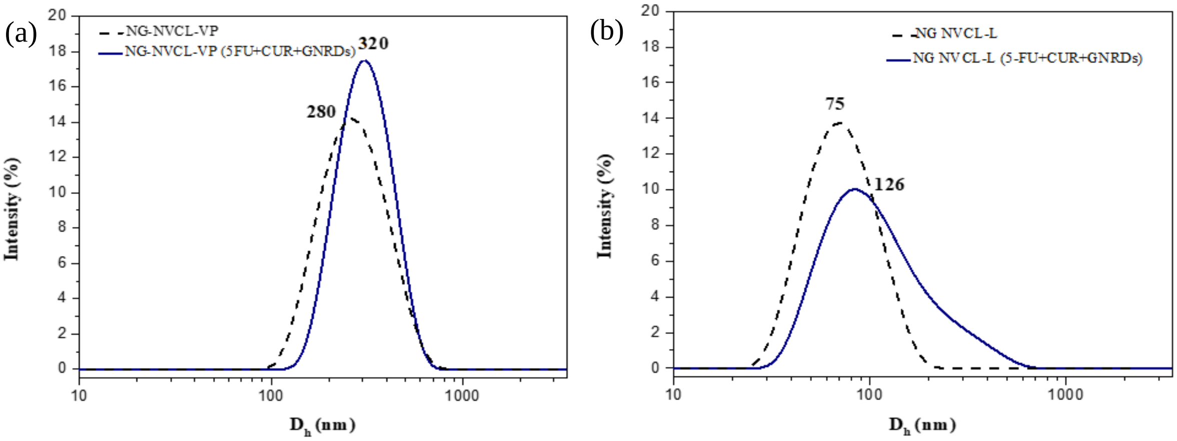

Figure S12. Size distribution by DLS in PBS 7.4 before and after concurrent loading of 5-FU, CUR and GNRDs of NG-NVCL-VP (a) and NG-NVCL-L (b).

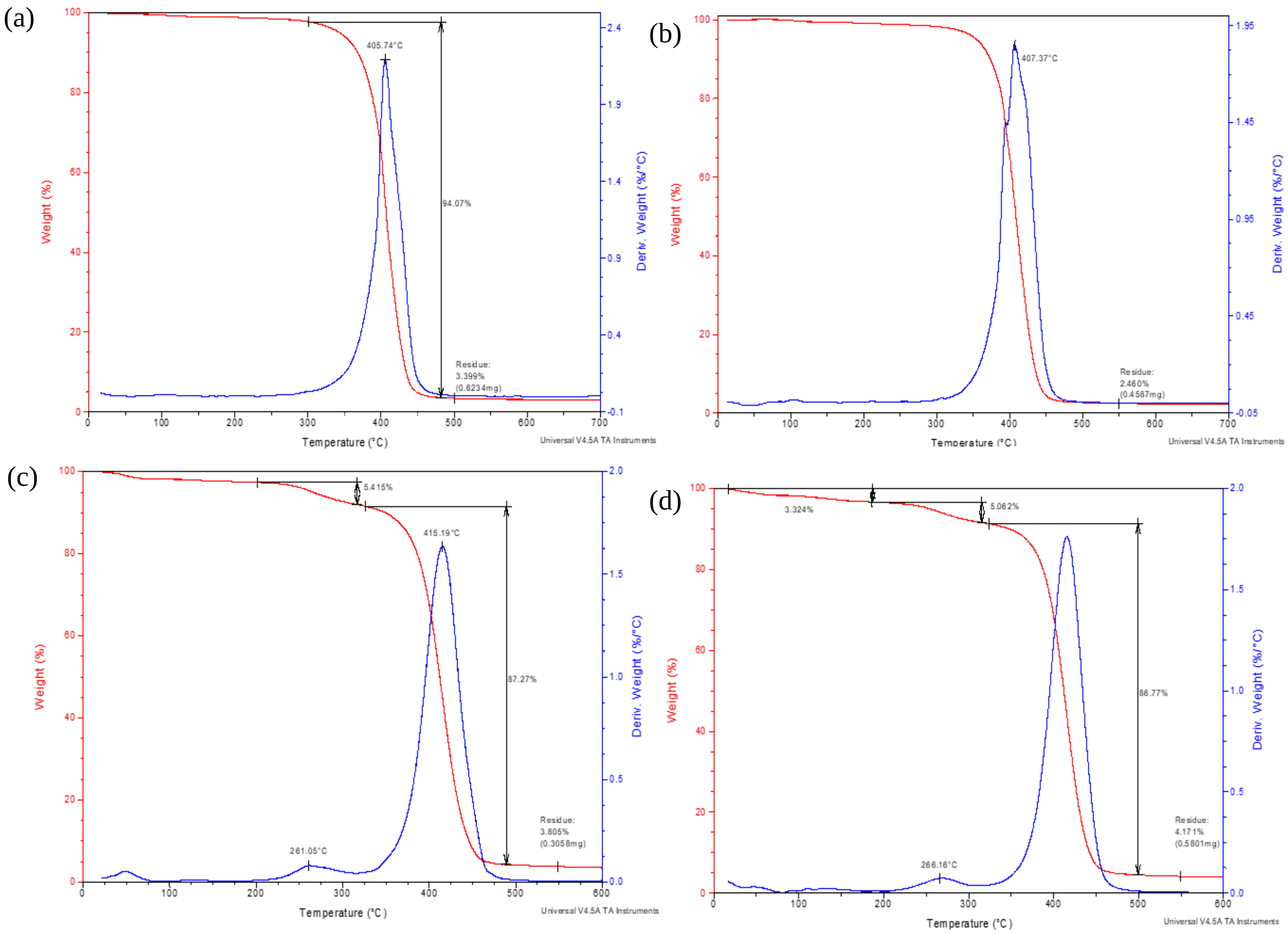

Figure S13. TGA thermogram of unloaded NGs: (a)NG-NVCL, (b)NG-NVCL-VP, (c)NG-NVCL-L and (d) NG-NVCL-L-VP.

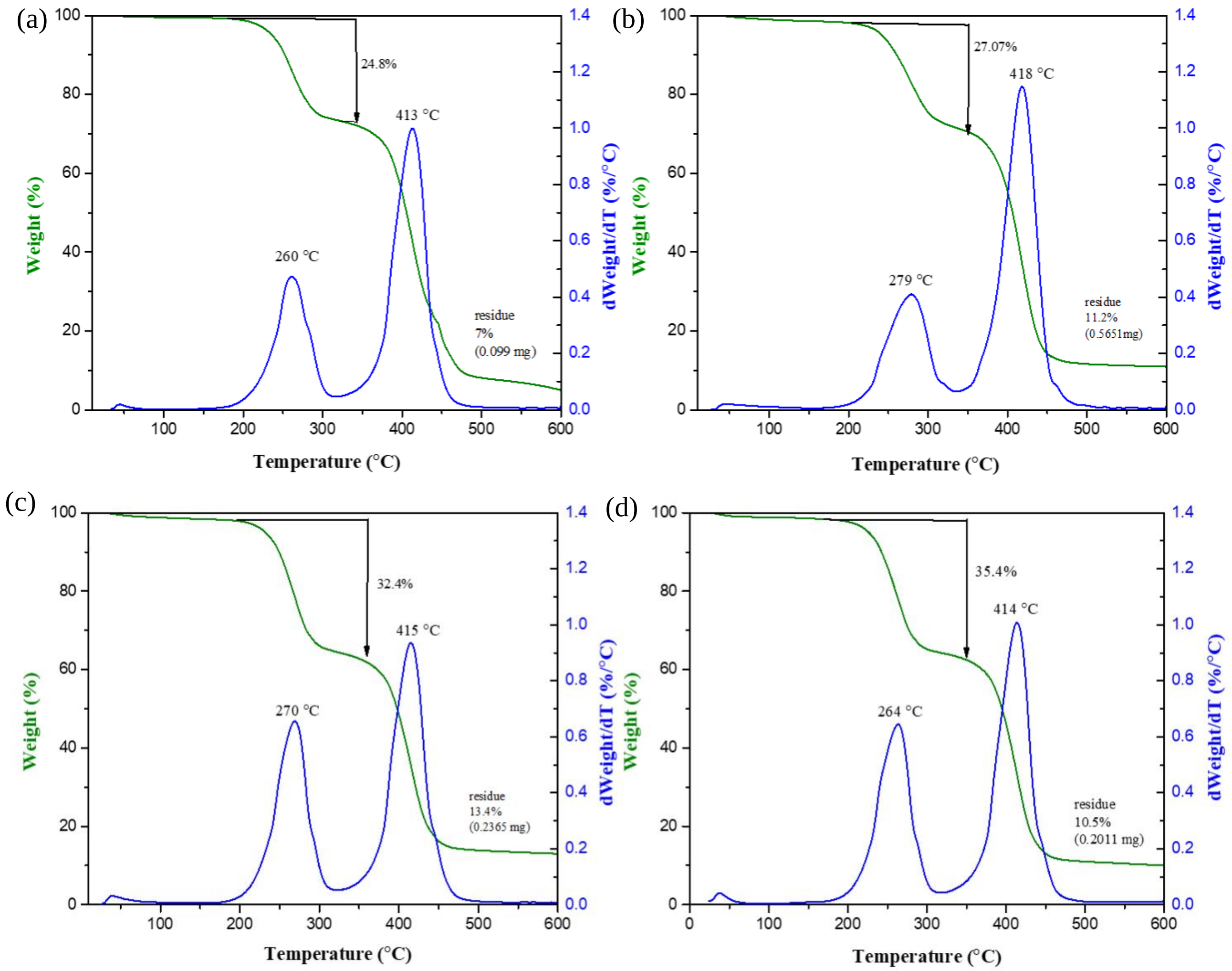

Figure S14. TGA thermograms of loaded NGs: (a)NG-NVCL (5-FU+CUR+GNRDs), (b)NG-NVCL-VP (5-FU+CUR+GNRDs), (c)NG-NVCL-L (5-FU+CUR+GNRDs) and (d) NG-NVCL-L-VP (5-FU+CUR+GNRDs).

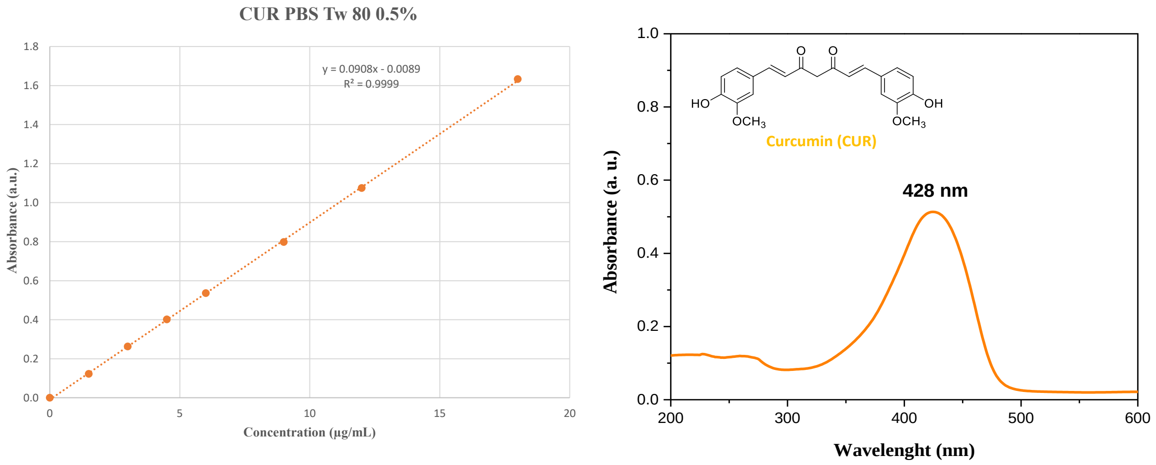

Figure S15. Calibration curve and UV-Vis spectrum of CUR in PBS with Tween 80 (0.5%).

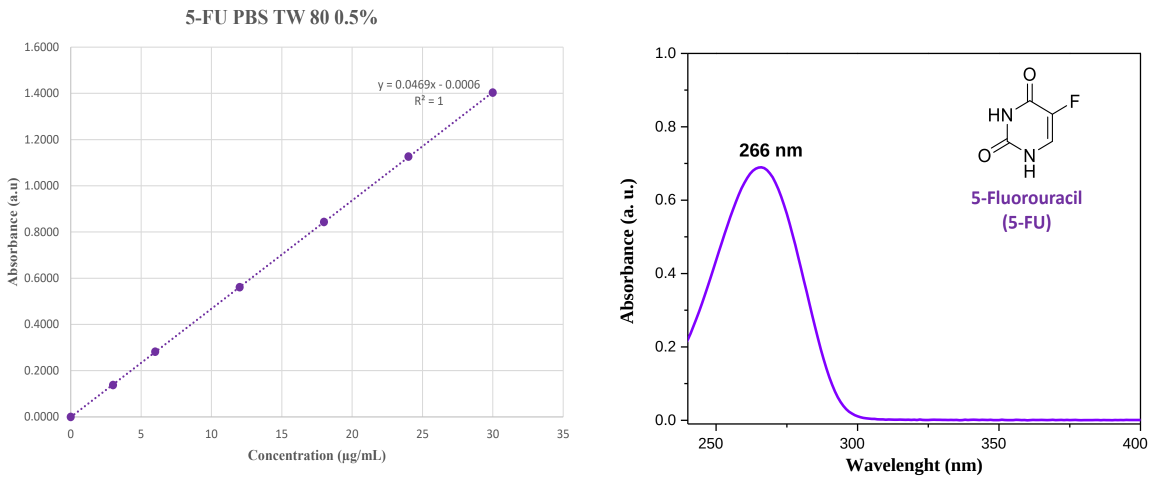

Figure S16. Calibration curve and UV-vis spectrum of 5-FU in PBS with Tween 80 (0.5%).

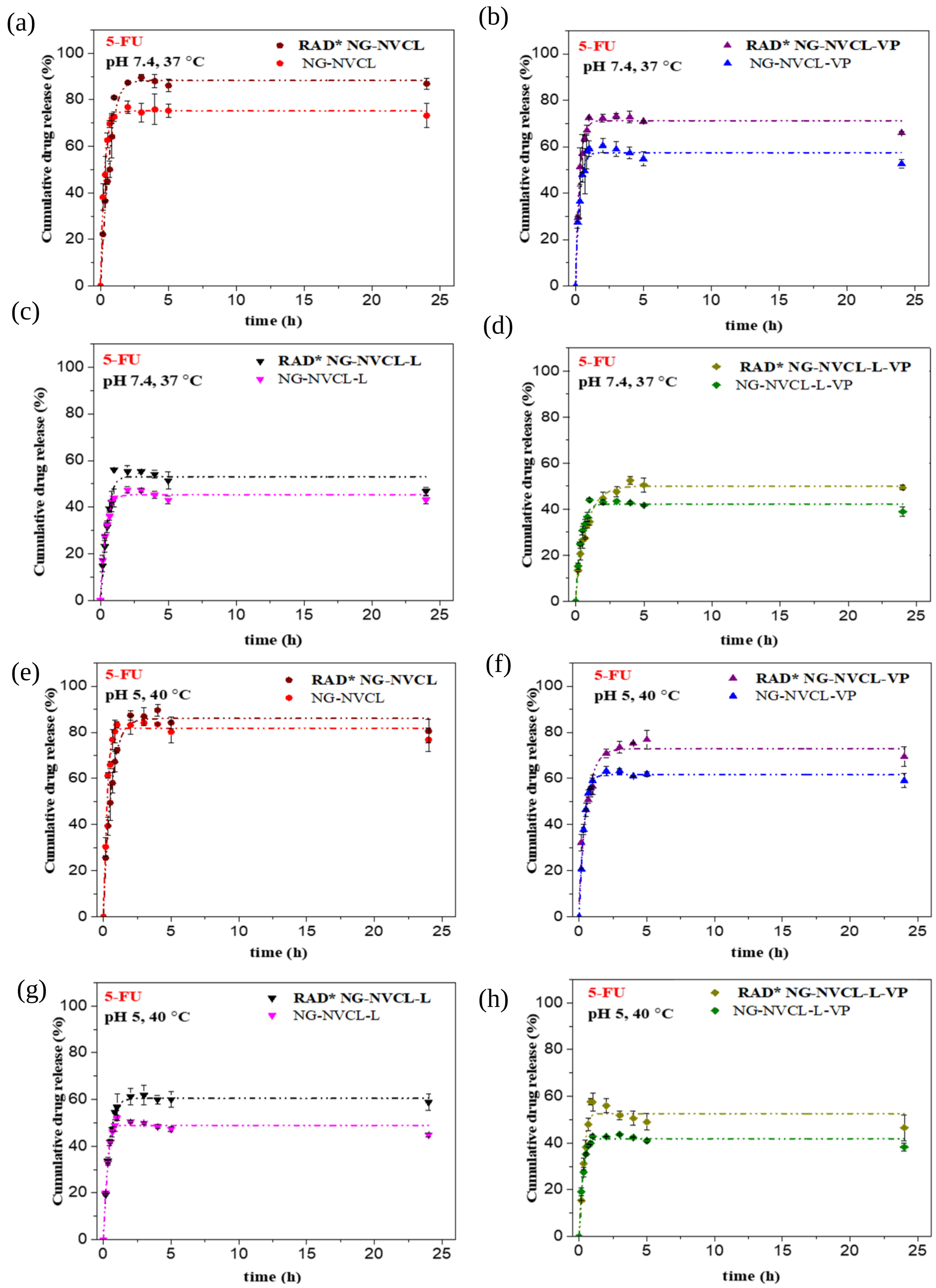

Figure S17. Release profiles of 5-FU at pH 7.4, 37 ºC (a, b, c, d) and at pH 5, 40 ºC (e, f, g, h) from NGs with/without concomitant NIR irradiation.

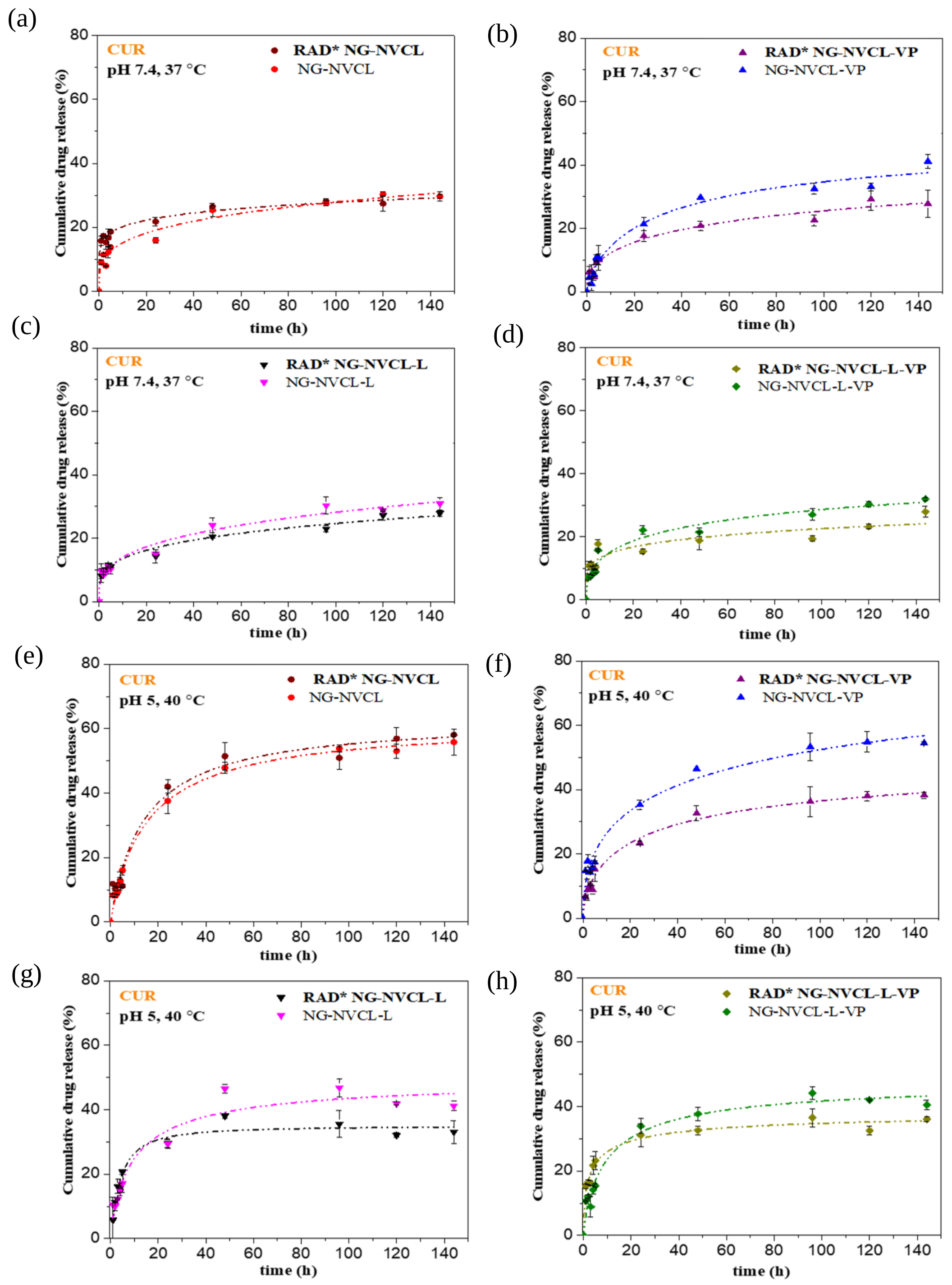

Figure S18. Release profiles of CUR at pH 7.4, 37 ºC (a, b, c, d) and at pH 5, 40 ºC (e, f, g, h) from NGs with/without concomitant NIR irradiation.

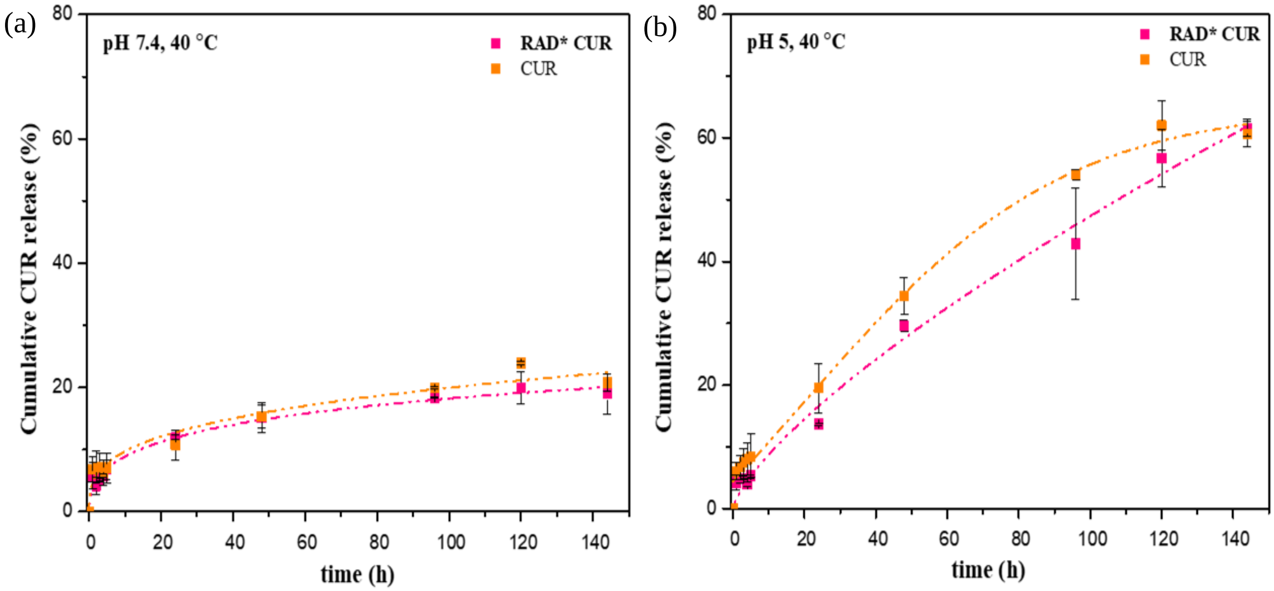

Figure S19. Release profiles of free CUR at pH 7.4, 37 ºC (a) and at pH 5, 40 ºC (b) with/without concomitant NIR irradiation.

Table S1. 5-FU *in vitro* release kinetics at pH 7.4, 37 °C: Data fitted with different mathematical models.

Model	Nanogel	5-FU pH 7.4, 37 °C			
		Without NIR	NIR	**Without NIR**	**NIR**
		k **(h^{-1})**		**r^2**	
**First order**	NG-NVCL	0.6645	1.020	0.69	0.98
	NG-NVCL-VP	0.4233	0.5957	0.67	0.67
	NG-NVCL-L	0.2959	0.4128	0.73	0.78
	NG-NVCL-L-VP	0.2629	0.2721	0.69	0.88
		k **($h^{-0.5}$)**		**r^2**	
**Higuchi**	NG-NVCL	0.5683	0.6767	0.85	0.95
	NG-NVCL-VP	0.4582	0.5504	0.95	0.85
	NG-NVCL-L	0.3614	0.4433	0.92	0.92
	NG-NVCL-L-VP	0.335	0.3248	0.90	0.98
		k **(h^{-n})**		**r^2**	
**Peppas**	NG-NVCL	0.7118<br>n= 0.31	0.6731<br>n= 0.58	0.88	0.95
	NG-NVCL-VP	0.556<br>n=0.35	0.6798<br>n= 0.36	0.95	0.83
	NG-NVCL-L	0.4147<br>n= 0.42	0.4566<br>n= 0.58	0.92	0.93
	NG-NVCL-L-VP	0.3842<br>n= 0.43	0.3423<br>n= 0.48	0.90	0.98

Table S2. CUR in vitro release kinetics at pH 7.4, 37 °C: Data fitted with different mathematical models.

Model	Nanogel	CUR pH 7.4, 37 °C Without NIR	NIR	Without NIR	NIR
		k **(h^{-1})**		**r^2**	
**First order**	NG-NVCL	0.0021	0.0015	0.84	0.64
	NG-NVCL-VP	0.0026	0.0014	0.84	0.77
	NG-NVCL-L	0.0022	0.0018	0.88	0.88
	NG-NVCL-L-VP	0.0023	0.0014	0.74	0.85
		k **($h^{-0.5}$)**		**r^2**	
**Higuchi**	NG-NVCL	0.0221	0.0166	0.92	0.73
	NG-NVCL-VP	0.0268	0.0162	0.93	0.87
	NG-NVCL-L	0.0235	0.0193	0.95	0.94
	NG-NVCL-L-VP	0.024	0.0153	0.78	0.93
		k **(h^{-n})**		**r^2**	
**Peppas**	NG-NVCL	0.0828 n=0.26	0.1473 n=0.14	0.92	0.95
	NG-NVCL-VP	0.0893 n= 0.29	0.0887 n= 0.20	0.95	0.95
	NG-NVCL-L	0.0740 n= 0.28	0.0783 n= 0.24	0.94	0.97
	NG-NVCL-L-VP	0.0679 n= 0.32	0.0982 n= 0.18	0.94	0.92
